# Structure-based drug design with equivariant diffusion models

**DOI:** 10.1038/s43588-024-00737-x

**Published:** 2024-12-09

**Authors:** Arne Schneuing, Charles Harris, Yuanqi Du, Kieran Didi, Arian Jamasb, Ilia Igashov, Weitao Du, Carla Gomes, Tom L. Blundell, Pietro Lio, Max Welling, Michael Bronstein, Bruno Correia

**Affiliations:** 1 École Polytechnique Fédérale de Lausanne, Lausanne, Switzerland; 2 University of Cambridge, Cambridge, UK; 3 Cornell University, Ithaca, NY USA; 4 Chinese Academy of Mathematics and System Science, Beijing, China; 5 University of Rome ‘La Sapienza’, Rome, Italy; 6 Microsoft Research AI4Science, Amsterdam, Netherlands; 7 University of Oxford, Oxford, UK; 8 AITHYRA Institute, Vienna, Austria; 9 Present Address: Prescient Design, Genentech, Basel, Switzerland; 10 Present Address: Heart and Lung Research Institute, University of Cambridge, Cambridge, UK; 11 Present Address: University of Amsterdam, Amsterdam, Netherlands

**Keywords:** Machine learning, Drug discovery

## Abstract

Structure-based drug design (SBDD) aims to design small-molecule ligands that bind with high affinity and specificity to pre-determined protein targets. Generative SBDD methods leverage structural data of drugs with their protein targets to propose new drug candidates. However, most existing methods focus exclusively on bottom-up de novo design of compounds or tackle other drug development challenges with task-specific models. The latter requires curation of suitable datasets, careful engineering of the models and retraining from scratch for each task. Here we show how a single pretrained diffusion model can be applied to a broader range of problems, such as off-the-shelf property optimization, explicit negative design and partial molecular design with inpainting. We formulate SBDD as a three-dimensional conditional generation problem and present DiffSBDD, an SE(3)-equivariant diffusion model that generates novel ligands conditioned on protein pockets. Furthermore, we show how additional constraints can be used to improve the generated drug candidates according to a variety of computational metrics.

## Abstract (web summary)

This work applies diffusion models to conditional molecule generation and shows how they can be used to tackle various structure-based drug design problems

## Main

The rational design of small molecules with drug-like properties remains an outstanding challenge in both fundamental and biopharmaceutical research. Structure-based drug design (SBDD) aims to find small-molecule ligands that bind to specific three-dimensional (3D) sites in proteins with high affinity and specificity^1^. Traditionally, SBDD campaigns are usually initiated either by high-throughput experimental or virtual screening^2,3^ of large chemical databases. In general, these approaches are expensive and time-consuming, but they also restrict the exploration of the chemical space to previously studied molecules, with a further emphasis usually placed on commercial availability^4^. Moreover, the optimization of initial lead molecules is often a biased process, with strong reliance on human intuition^5^. Recent advances in geometric deep learning, especially in modeling 3D structures of biomolecules^6–8^, provide a promising direction for SBDD^9^. Despite considerable progress in the use of deep learning as surrogate docking models^10–12^, deep learning-based design of ligands that bind to target proteins remains an overarching problem in molecular modeling. Early attempts have been made to represent molecules as atomic density maps, with variational autoencoders generating new atomic density maps corresponding to novel molecules^13^. However, it is non-trivial to map atomic density maps back to molecular space, requiring an additional atom-fitting stage. An alternative is to represent molecules as 3D graphs with atomic coordinates and types, which naturally circumvents the postprocessing steps. Li et al.^14^ proposed an autoregressive generative model to sample ligands given the protein pocket as a conditioning constraint. Peng et al.^15^ improved this method by using an E(3)-equivariant graph neural network (GNN), which respects rotation and translation symmetries in 3D space. Similarly, Drotár et al.^16^ and Liu et al.^17^ used autoregressive models to generate atoms sequentially and incorporate angles during the generation process. However, the main premise of sequential generation methods may not hold in real scenarios, as it imposes an artificial ordering scheme in the generation process and, as a result, the global context of the generated ligands may be lost. Very recently, a number of diffusion models have been put forward for target-specific molecule design^18–22^. These models place all atoms simultaneously, allowing them to reason about the whole molecule at once and typically enabling faster sampling. While this class of models has already shown great promise in de novo ligand generation, their potential in other parts of the drug design pipeline has not been thoroughly explored.

In this study, we propose DiffSBDD, an SE(3)-equivariant 3D conditional diffusion model for SBDD that respects translation, rotation and permutation symmetries. To evaluate our approach, we first show that diffusion models are a powerful framework for learning the distribution of 3D molecular data by generating new target-specific ligands de novo without additional constraints or optimizing a particular property (‘DiffSBDD captures the underlying data distribution’ section). We then demonstrate how the flexibility of diffusion models enables partial molecular redesign to incorporate specific design constraints without needing to develop specialized models (‘Generating chemical matter from known substructures’ section), and iterative improvement of molecular properties measured by user-specified oracles (‘Iterative search for better molecule candidates’ section). While we provide empirical results for only our model, the methodology can be readily used in combination with other recently published diffusion models for small-molecule design^18–22^.

## Equivariant diffusion models for SBDD

**Fig. 1:**
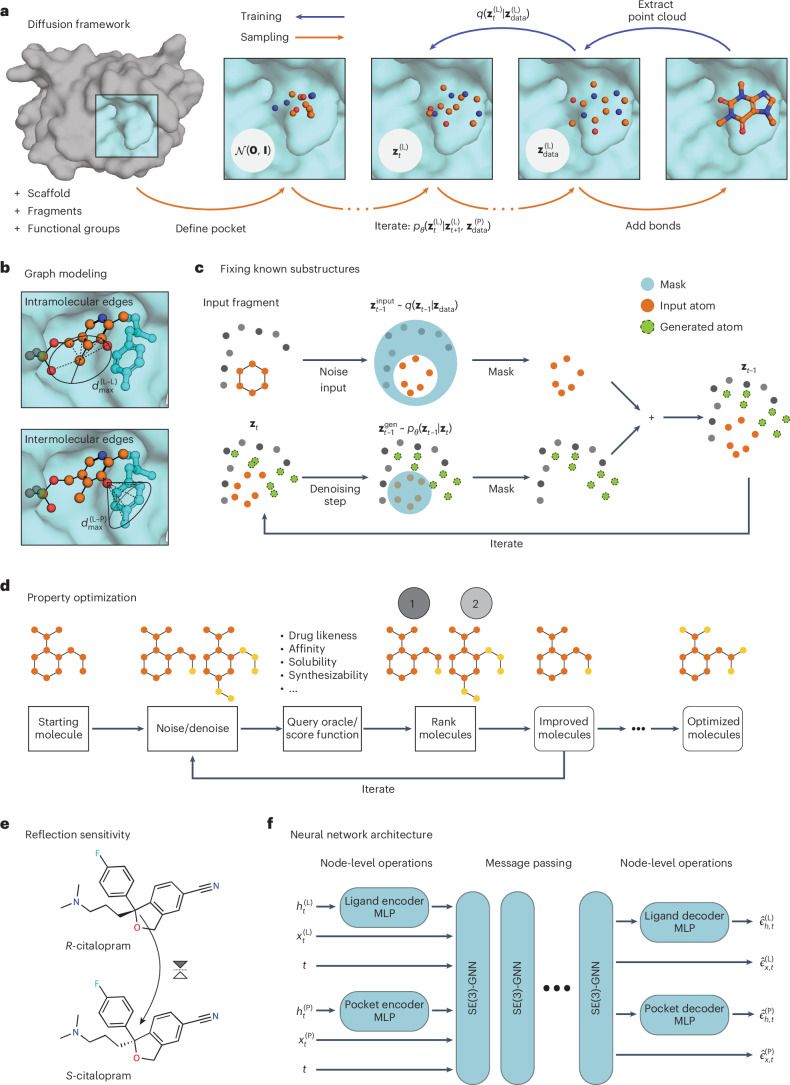
Method overview. **a**, The diffusion process *q* yields a noised version ${{\bf{z}}}_{t}^{({\mathrm{L}})}$ of the original atomic point cloud ${{\bf{z}}}_{{\rm{data}}}^{({\mathrm{L}})}$ for a time step *t* ≤ *T*. The neural network model is trained to approximate the reverse process conditioned on the target protein structure ${{\bf{z}}}_{{\rm{data}}}^{({\mathrm{P}})}$. Once trained, an initial noisy point cloud is sampled from a Gaussian distribution ${{\bf{z}}}_{T}^{({\mathrm{L}})} \sim {\mathcal{N}}\left({\bf{0}},{\bf{I}}\right)$ and progressively denoised using the learned transition probability *p*_*θ*_. Covalent bonds are added to the resultant point cloud at the end of the generation. Optionally, fixed substructures (for instance, fragments) can be provided to condition the generative process. Carbon, oxygen and nitrogen atoms are shown in orange, red and blue, respectively. **b**, Each state is processed as a graph where edges are introduced according to edge type-specific distance thresholds, for instance, ${d}_{\max }^{{\mathrm{L}}-{\mathrm{L}}}$ and ${d}_{\max }^{{\mathrm{L}}-{\mathrm{P}}}$. **c**, To generate new chemical matter conditioned on molecular substructures, we apply the learned denoising process to the entire molecule (superscript ‘gen’), but at every step we replace the prediction for the static substructure with the ground-truth noised version computed with *q* (superscript ‘input’). The protein context (gray) remains unchanged in every step. **d**, To tune molecular features, we find variations of a starting molecule by applying small amounts of noise and running an appropriate number of denoising steps. The new set of molecules is ranked by an oracle and the procedure is repeated for the best-scoring candidates. **e**, DiffSBDD is sensitive to reflections and can thus distinguish molecules with different stereochemistry. **f**, The neural network backbone is composed of MLPs that map scalar features *h* of ligand and pockets nodes into a joint embedding space, and SE(3)-equivariant message passing layers that operate on these features, each node’s coordinates *x* and a time step embedding *t*. It outputs the predicted noise values $\hat{\epsilon }$ for every vertex.

We leverage equivariant denoising diffusion probabilistic models (DDPMs)^23,24^ to generate molecules and binding conformations jointly for a given protein target. Figure 1a schematically depicts the 3D diffusion procedure. During training, varying amounts of random noise are applied to 3D structures of real ligands and a neural network learns to predict the noiseless features of the molecules. For sampling, these predictions are used to parameterize denoising transition probabilities, which allow us to gradually move a sample from a standard normal distribution onto the data manifold. Both the protein and the ligand are represented as 3D point clouds, where atom types are encoded as one-hot vectors and all objects are processed as graphs. For improved computational efficiency, we define independently tunable distance cut-offs for intermolecular edges between nodes of the ligand and pocket and intramolecular edges between two nodes from the same molecule (Fig. 1b). This means that information is propagated only between spatially proximal atoms. Our neural network is designed to respect natural symmetries of the molecular system, which include rotations and translations but exclude non-superposable transformations. That is, we process rigid transformations in an equivariant way but not reflections. This design choice is motivated by well-studied examples of drugs whose stereochemistry affects their activity and toxicity. For instance, the antidepressant citalopram (Fig. 1e) has two enantiomers but only the *S* enantiomer has the desired therapeutic effect. The difference between the *S* and *R* forms of the molecule, however, is only detectable by a reflection-sensitive GNN (Supplementary Section 4). Further technical details of the diffusion framework and equivariant neural network (Fig. 1f) are described in ‘Denoising diffusion probabilistic models’ and ‘SE(3)-equivariant GNNs’ in Methods.

To condition the 3D generative model on the structure of the protein pocket, we consider two distinct approaches. In the first approach, DiffSBDD-cond, we provide fixed 3D context in each step of the denoising process. To this end, we supplement the ligand atomic point cloud ${{\bf{z}}}_{t}^{({\mathrm{L}})}$, denoted by superscript L and diffusion time step *t*, with protein pocket nodes ${{\bf{z}}}_{{\rm{data}}}^{({\mathrm{P}})}$, denoted by superscript P, that remain unchanged throughout the reverse diffusion process (Fig. 1a). For the second method, DiffSBDD-joint, we initially train a diffusion model to approximate the joint distribution $p({{\bf{z}}}_{{\rm{data}}}^{({\mathrm{L}})},{{\bf{z}}}_{{\rm{data}}}^{({\mathrm{P}})})$ of ligand–pocket pairs, and inject information about target pockets only at inference time. The methodology is analogous to the substructure inpainting approach described below (‘Inpainting’ in Methods and Fig. 1c). Both approaches are equally applicable to the small-molecule design task and in practice differ in only whether the neural network expects the original pocket or a noisy version as input.

## DiffSBDD captures the underlying data distribution

As a first test to our model, we probe its ability to accurately represent the properties of real binders, and compare the results with Pocket2Mol^15^, ResGen^25^, PocketFlow^26^ and DeepICL^27^, four recently published autoregressive models, which represent the previous state-of-the-art class of machine learning models for SBDD. We use publicly available code and weights of the models (see ‘Code availability’). Note that not all baseline models have been trained on identical training sets (see ‘Experimental set-up’ in Methods).

**Fig. 2:**
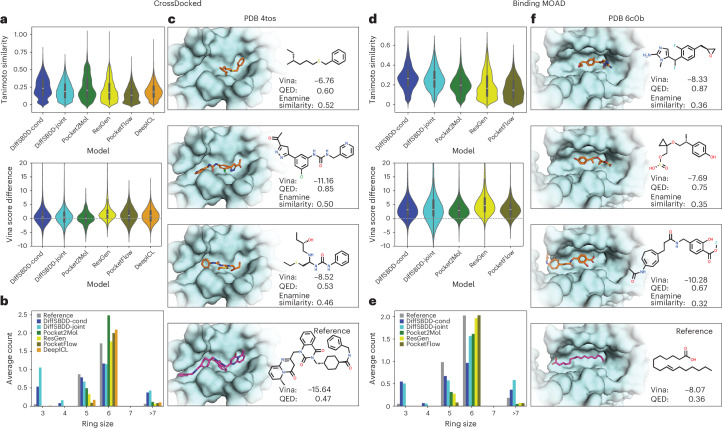
Evaluation of distribution learning capabilities and generated examples. All targets are taken from the CrossDocked and Binding MOAD test sets. **a**, Comparison of generated molecules with the reference molecule from the same pocket. We compare the Tanimoto similarity of the molecular fingerprints and compute the difference Vina_gen_ − Vina_ref_ between their Vina docking scores. *n* = 7,800, 7,800, 7,642, 8,932, 7,800 and 7,733, from left to right. **b**, Average number of rings of different sizes per generated molecule. **c**, Example molecules generated by DiffSBDD-cond for a pocket from the CrossDocked test set. We compared all generated molecules with the approximately 4.2 million compounds from the Enamine Screening Collection, and selected the three closest hits with drug-likeness QED > 0.5. Vina docking score, QED drug-likeness score and fingerprint similarity to the most similar Enamine molecules are reported in each case. **d**–**f**, The same analyses as in **a**–**c** but for target pockets from the Binding MOAD test set. *n* = 11,623, 11,581, 15,718, 13,072 and 11,900, from left to right. Carbon atoms are shown in orange or magenta. Oxygen, nitrogen, sulfur, chlorine and fluorine are shown in red, dark blue, yellow, green and light blue, respectively. All box plots within violins include the median line, a box denoting the interquartile range (IQR) and whiskers showing data within ±1.5 × IQR. Source data

Figure 2a shows that both DiffSBDD and Pocket2Mol Vina scores are centered around the reference but the spread is larger in the case of the diffusion models, which means that their samples contain larger fractions of low-scoring molecules but also ligands that potentially bind more tightly than the native counterparts. The greater abundance of high-scoring molecules is particularly important in anticipation of downstream design applications, where we often look for the most competitive binder rather than average candidates. A similar observation holds for the Binding MOAD^28^ dataset with experimentally determined binding complexes. However, unlike the CrossDocked case, docking scores are worse on average than the scores of corresponding reference ligands from this dataset. We believe the reason to be twofold: the Binding MOAD training set is much smaller and also contains more challenging ground-truth ligands (native binders) whereas CrossDocked complexes can have unrealistic protein–ligand interactions. This hypothesis is supported by less favorable Vina scores of reference molecules from the synthetic dataset on average (−7.68 versus −9.17). This result underscores the importance of high-quality training sets for SBDD models that aim to design high-affinity binders. Lastly, the DiffSBDD models also produce molecules that are slightly more similar to the reference on average (Fig. 2a,d) and contain a comparable amount of five- and six-membered rings to natural ligands (Fig. 2b,e). However, very small and very large ring systems consisting of less than four or more than seven atoms, respectively, are typically over-represented in DiffSBDD molecules. It is worth mentioning that differences between the two conditioning approaches (DiffSBDD-cond and DiffSBDD-joint) are typically much smaller than the differences between DiffSBDD and other models. Thus, the empirical evidence does not clearly favor one conditioned diffusion approach over the other. Additional tests of the distribution learning capabilities are summarized in Supplementary Section 5.1.

Figure 2c,f shows a representative selection of molecules for one target from each test set. The selection is filtered to contain examples that are drug-like (quantitative estimate of drug-likeness (QED) > 0.5) and similar to purchasable molecules from the Enamine Screening Collection. These filters represent favorable properties one might look for in a drug design campaign. The target with Protein Data Bank (PDB) identifier 6c0b, for example, is a human receptor that is involved in microbial infection^29^ and possibly tumor suppression^30^. The reference molecule, a long fatty acid (Fig. 2f, bottom) that aids receptor binding^29^, has too high a number of rotatable bonds and low a number of hydrogen bond donors/acceptors to be considered a suitable drug-like compound (QED = 0.36). Our model, however, generates drug-like (QED = 0.87 in the first example) and suitably sized molecules by adding aromatic rings connected by a few rotatable bonds, which allows the molecules to adopt a complementary binding geometry and is entropically favorable by reducing the degrees of freedom, a classic approach in medicinal chemistry^31^. Larger random samples of generated molecules are presented in Supplementary Figs. 8 and 9. Moreover, Supplementary Table 4 summarizes the fractions of novel and unique generated molecules.

## Generating chemical matter from known substructures

**Extended Data Fig. 1:**
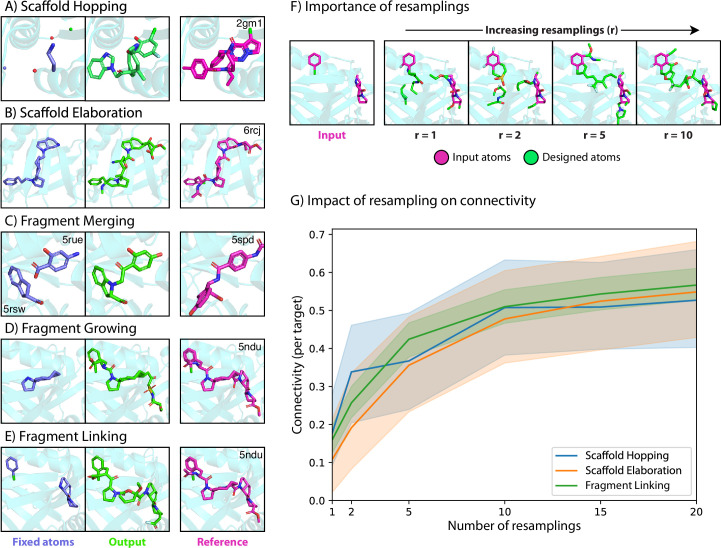
Molecular inpainting results. Design examples for scaffold hopping (**A**), scaffold elaboration (**B**), fragment merging (**C**), fragment growing (**D**) and fragment linking (**E**). The inputs to our model (the fixed atoms) are shown in blue, the outputs (designed molecules) are shown in green and the original molecules are shown in magenta for reference. PDB codes are shown for the ground truth structure. In the case of fragment merging, we compose fragments with two different crystal structures with PDB codes shown. (**F**) Importance of resampling for generating realistic and connected molecules. The designed region (green) finally harmonizes with the molecular context at high resamplings. (**G**) Effect of the number of resampling steps on molecular connectivity. Carbon atoms are shown in light blue, green, or magenta depending on atom character. Oxygen, nitrogen, sulfur and chlorine are shown in red, dark blue, yellow, and light green, respectively. Means and 95% confidence intervals are plotted for 3 design tasks. For this experiment we used 20 randomly selected targets from the test set. Source data

In drug discovery, it is common to design molecules around previously identified active substructures. For example, some important tasks are to design a scaffold around a set of functional groups (scaffold hopping) or extend an existing fragment to make a whole molecule (fragment growing). Generating compounds, or parts thereof, conditioned on a given molecular context is reminiscent of inpainting, a technique originally introduced for completing missing parts of images^32,33^ but also adopted in other domains, including biomolecular structures^34^. We can realize a number of drug discovery sub-tasks via an inpainting technique known as the ‘replacement method’^33,35^, whereby we add new atoms in and around fixed regions of the substructure to design whole molecules (Fig. 1c and ‘Inpainting’ in Methods). Unlike previous methods, using DiffSBDD in this way does not require retraining a model on any specialized or synthetic datasets. Curating such datasets is often time and labor intensive, and typically relies on potentially sub-optimal assumptions (for example, definition of fragments) to convert a general dataset of small molecules into a task-specific dataset that can be used to train specialized models. With our proposed approach, by contrast, the simple definition of an arbitrary binary mask is sufficient for the diffusion model to generalize to any inpainting task while using a neural network trained on all available protein–ligand data in raw form. Examples of five different design applications can be found in Extended Data Fig. 1. A systematic test on the Binding MOAD test set in the tasks of linker design, scaffold hopping and scaffold elaboration is presented in Supplementary Section 5.5. We find that constraining fixed regions to highly complementary substructures within the protein pocket substantially enhances Vina scores compared with the baseline version of DiffSBDD in all three tasks. For fragment linking, our general sampling strategy even achieves results comparable to the specialist model DiffLinker^36^.

## Iterative search for better molecule candidates

**Extended Data Fig. 2:**
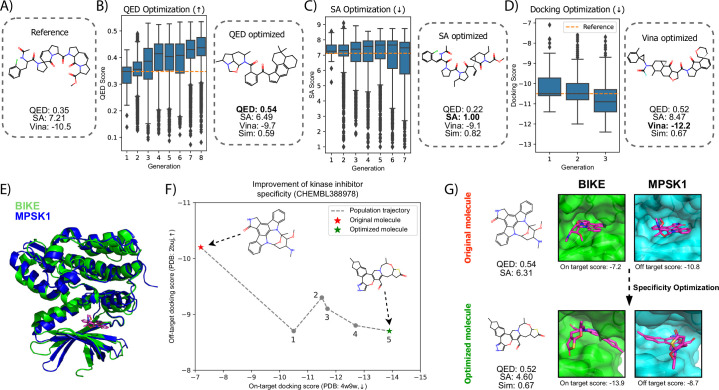
Results on molecular optimization using DiffSBDD. (**A**-**D**) Experiments on single property molecular optimization. (**A**) Starting inhibitor from PDB code 5ndu. (**B**) QED optimization over 8 generations. (**C**) SA optimization over 7 generations. (**D**) docking score optimization over 3 generations. We found that optimization over subsequent generations continuously optimized the docking score, but that was at expense of molecular quality. (**E**-**G**) Kinase inhibitor specificity optimization experiment. (**E**) Cartoon representation showing the high degree of structural similarity between our two kinases of interest (BIKE and MPSK1). (**F**) Trajectory plot showing the highest scoring molecule at each iteration during kinase inhibitor optimization. (**G**) Visual representation of the molecular graphs and bound conformations of the native and final molecules with corresponding Vina docking scores. Boxes in panels (**B**-**D**) represent the upper and lower quartile as well as the median of the data. Whiskers denote 1.5 times the interquartile range. Outliers outside this range are shown as flier points. Sample sizes for each generation are 80, 4474, 4390, 4460, 4459, 4470, 4472, 4474 for panel B, 84, 4500, 4500, 4500, 4500, 4500, 4500 for panel C and 118, 432, 437 for panel D. Carbon, oxygen, nitrogen and sulfur are shown in magenta, red, dark blue and yellow, respectively. QED: Quantitative Estimation of Drug-likeness; SA: Synthetic Accessibility; Sim.: Tanimoto molecular fingerprint similarity to the reference. Source data

For hit identification and optimization of lead molecules in real use cases, it is not enough to just sample molecules from the whole training data distribution. Instead, we are usually interested in the better-performing tail of the distribution, and only want to pursue the most promising candidates. As we could show that DiffSBDD recapitulates the chemical space of the training set including high-scoring molecules, we should always find promising drug candidates with strong docking scores, synthetic accessibility and other desired properties. Here we propose a simple protocol to access them efficiently through repeated noising/denoising combined with selection of the most promising candidates in each iteration (Fig. 1d and ‘Implementation details’ in Methods). Optimization of synthetic accessibility, QED and Vina scores is demonstrated in Extended Data Fig. 2a–d.

Furthermore, we consider the challenging case of highly selective kinase inhibitor design (Extended Data Fig. 2e–g). In our experiment, we perform positive design against our on-target kinase BIKE (PDB code 4w9w) while simultaneously performing negative design against the structurally similar off-target kinase MPSK1 (PDB code 2buj) (Extended Data Fig. 2e). Within five rounds of optimization, we managed to improve the on-target docking score from −7.2 to −13.9 while simultaneously decreasing the off-target value from −10.8 to −8.7, demonstrating substantially improved specificity.

## Conclusion

Many machine learning methods for SBDD focus exclusively on the de novo generation of new ligands from scratch, which often limits their sample quality and synthesizability, and ultimately hinders lab validation of designs. While the purely de novo design of chemical matter remains challenging for our diffusion model, we could show that learning-based tools are ready to be incorporated in drug development pipelines if additional design constraints are enforced. Constraining the problem to realistic substructures such as fragments or scaffolds leads to better designs because it prevents the neural network from overly hallucinating. Retaining substructures of previously synthesized molecules holds promise in facilitating chemical synthesis and experimental testing. Moreover, the capability to further ‘locally’ (in chemical space) optimize designed ligands is important in real-world drug discovery and effectively improves the quality of the initial designs. For similar applications, previous studies typically resorted to specialized models that were trained on tailored datasets and performed well on only narrowly defined tasks. Here we provided evidence that a powerful general diffusion model can be used as a drop-in replacement for these specialized models if the sampling procedure is modified appropriately. This means in the future we can expect better performance in all discussed sub-tasks, solely by improving the distribution learning capabilities and sample quality of the main model.

## Methods

### Denoising diffusion probabilistic models

DDPMs^23^ are a class of generative models inspired by non-equilibrium thermodynamics. In brief, they define a Markovian chain of random diffusion steps by slowly adding noise to sample data and then learning the reverse of this process (typically via a neural network) to reconstruct data samples from noise.

In this work, we closely follow the framework developed by Hoogeboom et al.^24^. In our setting, data samples are atomic point clouds **z**_data_ = [**x**, **h**] with 3D geometric coordinates ${\bf{x}}\in {{\mathbb{R}}}^{N\times 3}$ and categorical features ${\bf{h}}\in {{\mathbb{R}}}^{N\times d}$, where *N* is the number of atoms. A fixed noise process

1 $$q\left({{\bf{z}}}_{t}| {{\bf{z}}}_{{\rm{data}}}\right)={\mathcal{N}}\left({{\bf{z}}}_{t}| {\alpha }_{t}{{\bf{z}}}_{{\rm{data}}},{\sigma }_{t}^{2}{I}\right)$$

adds noise to the data **z**_data_ and produces a latent noised representation **z**_*t*_ for *t* = 0, …, *T*. ${\sigma }_{t}^{2}$ is the variance of the Gaussian noise distribution. *α*_*t*_ controls the signal-to-noise ratio $\,\text{SNR}\,(t)={\alpha }_{t}^{2}/{\sigma }_{t}^{2}$ and follows either a learned or pre-defined schedule from *α*_0_ ≈ 1 to *α*_*T*_ ≈ 0 (ref. ^37^). We choose a variance-preserving noising process^32^ with ${\alpha }_{t}=\sqrt{1-{\sigma }_{t}^{2}}$. *I* is an identity matrix.

As the noising process is Markovian, we can write the denoising transition from time step *t* to *s* < *t* in closed form as

2 $$q({{\bf{z}}}_{s}| {{\bf{z}}}_{{\rm{data}}},{{\bf{z}}}_{t})={\mathcal{N}}\left({{\bf{z}}}_{s}\left\vert \frac{{\alpha }_{t| s}{\sigma }_{s}^{2}}{{\sigma }_{t}^{2}}\right.{{\bf{z}}}_{t}+\frac{{\alpha }_{s}{\sigma }_{t| s}^{2}}{{\sigma }_{t}^{2}}{{\bf{z}}}_{{\rm{data}}},\frac{{\sigma }_{t| s}^{2}{\sigma }_{s}^{2}}{{\sigma }_{t}^{2}}{I}\right)$$

with ${\alpha }_{t| s}=\frac{{\alpha }_{t}}{{\alpha }_{s}}$ and ${\sigma }_{t| s}^{2}={\sigma }_{t}^{2}-{\alpha }_{t| s}^{2}{\sigma }_{s}^{2}$ following the notation of Hoogeboom et al.^24^. This true denoising process depends on the data sample **z**_data_, which is not available when using the model for generating new samples. Instead, a neural network *ϕ*_*θ*_, where *θ* indicates trainable parameters, is used to approximate the sample ${\hat{{\bf{z}}}}_{{\rm{data}}}$. More specifically, we can reparameterize equation (1) as **z**_*t*_ = *α*_*t*_**z**_data_ + *σ*_*t*_**ϵ** with ${\bf{\upepsilon }} \sim {\mathcal{N}}({\bf{0}},{I})$ and directly predict the Gaussian noise ${\hat{{\bf{\upepsilon }}}}_{\theta }={\phi }_{\theta }({{\bf{z}}}_{t},t)$. Thus, ${\hat{{\bf{z}}}}_{{\rm{data}}}$ is simply given as ${\hat{{\bf{z}}}}_{{\rm{data}}}=\frac{1}{{\alpha }_{t}}{{\bf{z}}}_{t}-\frac{{\sigma }_{t}}{{\alpha }_{t}}{\hat{{\bf{\upepsilon }}}}_{\theta }$.

The neural network is trained to maximize the likelihood of observed data by optimizing a variational lower bound on the data, which is equivalent to the simplified training objective ${{\mathcal{L}}}_{{\rm{train}}}=\frac{1}{2}| | {\bf{\upepsilon }}-{\phi }_{\theta }({{\bf{z}}}_{t},t)| {| }^{2}$ up to a scale factor^23,37^. See Supplementary Section 1 for details.

### Equivariance

Structural biology remains a rather data-sparse domain. It is therefore common practice to encode known geometric constraints, typically equivariance to rotations and translations, directly into the neural network architecture, thereby facilitating the learning task because possible neural operations are limited to a meaningful subset. In the 3D molecule-generation setting, we explicitly exclude reflection-equivariant operations because they would make the model blind to some aspects of stereochemistry. It is known that different stereoisomers can have fundamentally different therapeutic effects (for example, ref. ^38^; Fig. 1e) and might even lead to unforeseen off-target activity and hence toxicity. We therefore developed a reflection-sensitive system that is SE(3)-equivariant rather than E(3)-equivariant although the latter is more commonly adopted in related studies^18,24,39^.

Technically, we ensure SE(3)-equivariance in the following sense: evaluating the likelihood of a molecule ${{\bf{x}}}^{({\mathrm{L}})}\in {{\mathbb{R}}}^{3\times {N}_{{\mathrm{L}}}}$ given the 3D representation of a protein pocket ${{\bf{x}}}^{({\mathrm{P}})}\in {{\mathbb{R}}}^{3\times {N}_{{\mathrm{P}}}}$ should not depend on global SE(3)-transformations of the system, meaning *p*(*R***x**^(L)^ + **t**∣*R***x**^(P)^ + **t**) = *p*(**x**^(L)^∣**x**^(P)^) for orthogonal ${R}\in {{\mathbb{R}}}^{3\times 3}$ with *R*^*T*^*R* = *I*, $\det ({R})=1$ and ${\bf{t}}\in {{\mathbb{R}}}^{3}$ added column-wise. At the same time, it should be possible to generate samples **x**^(L)^ ~ *p*(**x**^(L)^∣**x**^(P)^) from this conditional probability distribution so that equivalently transformed ligands *R***x**^(L)^ + **t** are sampled with the same probability if the input pocket is rotated and translated and we sample from *p*(*R***x**^(L)^ + **t**∣*R***x**^(P)^ + **t**). This definition explicitly excludes reflections that are connected with chirality and can alter the biomolecule’s properties. Node-type features, which transform invariantly, are ignored in this discussion for simpler notation.

In our set-up, equivariance to the orthogonal group *O*(3) (comprising rotations and reflections) is achieved because we model both prior and transition probabilities with isotropic Gaussians where the mean vector transforms equivariantly with respect to rotations of the context (see Hoogeboom et al.^24^ and Supplementary Section 3). Ensuring translation equivariance, however, is harder because the transition probabilities *p*(**z**_*t*−1_∣**z**_*t*_) are not inherently translation-equivariant. To circumvent this issue, we follow previous studies^24,40^ by limiting the whole sampling process to a linear subspace where the center of mass (COM) of the system is zero. In practice, this is achieved by subtracting the COM of the system before performing likelihood computations or denoising steps. As equivariance of the transition probabilities depends on the parameterization of the noise predictor ${\hat{{\bf{\upepsilon }}}}_{\theta }$, we can make the model sensitive to reflections with a simple additive cross-product term in the neural network’s coordinate update as discussed in the next section and Supplementary Section 4.

### SE(3)-equivariant GNNs

A function $f:{\mathcal{X}}\to {\mathcal{Y}}$ is said to be equivariant with respect to the group *G* if *f*(*g*.**x**) = *g*.*f*(**x**), where *g*. denotes the action of the group element *g* ∈ *G* on ${\mathcal{X}}$ and ${\mathcal{Y}}$ (ref. ^41^). GNNs are learnable functions that process graph-structured data in a permutation-equivariant way, making them particularly useful for molecular systems where nodes do not have an intrinsic order. Permutation invariance means that GNN(*ΠX*) = *Π*GNN(*X*) where *Π* is an *n* × *n* permutation matrix acting on the node feature matrix.

As the nodes of the molecular graph represent the 3D coordinates of atoms, we are interested in additional equivariance with respect to the Euclidean group E(3) or rigid transformations. An E(3)-equivariant GNN (EGNN) satisfies EGNN(*ΠXA* + **b**) = *Π* EGNN(*X*)*A* + **b** for an orthogonal 3 × 3 matrix *A* with *A*^⊤^*A* = *I* and some translation vector **b** added row-wise.

In our case, as the nodes have both geometric atomic coordinates **x** as well as atomic type features **h**, we can use a simple implementation of EGNN proposed by Satorras et al.^39^, in which the updates for features **h** and coordinates **x** of node *i* at layer *l* are computed as follows:

3 $${{\bf{m}}}_{ij}={\phi }_{e}\left({{\bf{h}}}_{i}^{l},{{\bf{h}}}_{j}^{l},{d}_{ij}^{\,2},{a}_{ij}\right),\,{\tilde{e}}_{ij}={\phi }_{{\rm{att}}}\left({{\bf{m}}}_{ij}\right)$$

4 $${{\bf{h}}}_{i}^{l+1}={\phi }_{h}\left({{\bf{h}}}_{i}^{l},\sum _{j\ne i}{\tilde{e}}_{ij}{{\bf{m}}}_{ij}\right)$$

5 $${{\bf{x}}}_{i}^{l+1}={{\bf{x}}}_{i}^{l}+\sum _{j\ne i}\frac{{{\bf{x}}}_{i}^{l}-{{\bf{x}}}_{j}^{l}}{{d}_{ij}+1}{\phi }_{x}\left({{\bf{h}}}_{i}^{l},{{\bf{h}}}_{j}^{l},{d}_{ij}^{2},{a}_{ij}\right)$$

where *ϕ*_*e*_, *ϕ*_att_, *ϕ*_*h*_ and *ϕ*_*x*_ are learnable multilayer perceptrons (MLPs) and *d*_*ij*_ and *a*_*ij*_ are the relative distances and edge features between nodes *i* and *j* respectively. *m*_*ij*_ and $\tilde{e}_{ij}$ are messages and attention coefficients, respectively. Following Igashov et al.^36^, we do not update the coordinates of nodes that belong to the pocket to ensure the 3D protein context remains fixed throughout the EGNN layers.

We can break the symmetry to reflections and thereby make the GNN layer SE(3)-equivariant by adding a cross-product-dependent term to the coordinate update, which changes sign under reflection:

6 $${{\bf{x}}}_{i}^{l+1}={{\bf{x}}}_{i}^{l}+\sum _{j\ne i}\frac{{{\bf{x}}}_{i}^{l}-{{\bf{x}}}_{j}^{l}}{{d}_{ij}+1}{\phi }_{x}^{d}\left({{\bf{h}}}_{i}^{l},{{\bf{h}}}_{j}^{l},{d}_{ij}^{2},{a}_{ij}\right)$$

7 $$+\frac{\left({{\bf{x}}}_{i}^{l}-{\bar{{\bf{x}}}}^{l}\right)\times \left({{\bf{x}}}_{j}^{l}-{\bar{{\bf{x}}}}^{l}\right)}{\parallel \left({{\bf{x}}}_{i}^{l}-{\bar{{\bf{x}}}}^{l}\right)\times \left({{\bf{x}}}_{j}^{l}-{\bar{{\bf{x}}}}^{l}\right)\parallel +1}{\phi }_{x}^{\times }\left({{\bf{h}}}_{i}^{l},{{\bf{h}}}_{j}^{l},{d}_{ij}^{2},{a}_{ij}\right).$$

Here, ${\bar{{\bf{x}}}}^{l}$ denotes the COM of all nodes at layer *l*. ${\phi }_{x}^{\times }$ is an additional MLP. The desired SE(3)-equivariance of this modification is discussed in Supplementary Section 4.

### Inpainting

For molecular inpainting as shown in Fig. 1c, a subset of all atoms is fixed and serves as the molecular context we want to condition on. All other atoms are generated by the DDPM. To this end, we sample a diffused representation ${{\bf{z}}}_{t}^{{\rm{input}}}$ of the fixed atoms **z**_data_ at every time step *t* in addition to the predicted latent representation ${z}_{t}^{\,{\rm{gen}}}$. A set of mask indices ${\mathcal{M}}$ uniquely identifies nodes corresponding to fixed atoms in ${{\bf{z}}}_{t}^{\,{\rm{gen}}}$. Note that ${{\bf{z}}}_{t}^{{\rm{input}}}$ contains exactly $| {\mathcal{M}}|$ atoms while ${z}_{t}^{\,{\rm{gen}}}$ is bigger. For every denoising step, we then replace the generated atoms corresponding to fixed nodes (${{\bf{z}}}_{t-1,i\in {\mathcal{M}}}^{\,{\rm{gen}}}$) with their forward noised counterparts:

8 $${{\bf{z}}}_{t-1}^{{\rm{input}}} \sim q\left({{\bf{z}}}_{t-1}| {{\bf{z}}}_{{\rm{data}}}\right)$$

9 $${{\bf{z}}}_{t-1}^{{\rm{gen}}} \sim {p}_{\theta }\left({{\bf{z}}}_{t-1}| {{\bf{z}}}_{t}\right)$$

10 $${{\bf{z}}}_{t-1}=\left[{{\bf{z}}}_{t-1}^{{\rm{input}}},{{\bf{z}}}_{t-1,i\notin {\mathcal{M}}}^{{\rm{gen}}}\right].$$

In this manner, we traverse the Markov chain in reverse order from *t* = *T* to *t* = 0 to generate conditional samples. Because the noise schedule decreases the noising process’s variance to almost zero at *t* = 0 (‘Denoising diffusion probabilistic models’ section), the final sample is guaranteed to contain an unperturbed representation of the fixed atoms. This approach was applied to pocket-conditioned ligand inpainting by fixing all pocket nodes when sampling from the joint distribution model (DiffSBDD-joint). It was also used in the substructure design experiments.

#### Equivariance

As the equivariant diffusion process is defined for a COM-free system, we must ensure that this requirement remains satisfied after the substitution step in equation (10). To prevent a COM shift, we therefore translate the fixed atom representation so that its COM coincides with the predicted representation:

11 $${\tilde{{\bf{x}}}}_{t-1}^{{\rm{input}}}={{\bf{x}}}_{t-1}^{{\rm{input}}}+\frac{1}{n}\sum _{i\in {\mathcal{M}}}{{\bf{x}}}_{t-1,i}^{{\rm{gen}}}-\frac{1}{n}\sum _{i\in {\mathcal{M}}}{{\bf{x}}}_{t-1,i}^{{\rm{input}}}$$

before creating the new combined representation

12 $${{\bf{z}}}_{t-1}=\left[{\tilde{{\bf{z}}}}_{t-1}^{{\rm{input}}},{{\bf{z}}}_{t-1,i\notin {\mathcal{M}}}^{{\rm{gen}}}\right]$$

with $${\tilde{{\bf{z}}}}_{t-1}^{{\rm{input}}}=\left[{\tilde{{\bf{x}}}}_{t-1}^{{\rm{input}}},{{\bf{h}}}_{t-1}^{{\rm{input}}}\right]$$ and $$n=| {\mathcal{M}}|$$.

#### Resampling

Trippe et al.^42^ showed that this simple replacement method inevitably introduces approximation error that can lead to inconsistent inpainted regions. In our experiments, we observe that the inpainting solution sometimes generates disconnected molecules that are not properly positioned in the target pocket (see Supplementary Fig. 1a for an example). Trippe et al.^42^ proposed to address this limitation with a particle filtering scheme that upweights more consistent samples in each denoising step. We, however, choose to adopt the conceptually simpler idea of resampling^33^, where each latent representation is repeatedly diffused back and forth before advancing to the next time step as demonstrated in the algorithm in Supplementary Section 6.4. This enables the model to harmonize its prediction for the generated part and the noisy sample from the fixed part, which does not include any information about the generated part. We choose *r* = 10 resamplings per denoising step for our experiments with DiffSBDD-joint based on empirical results discussed in Supplementary Section 5.4.

### Implementation details

#### Molecule size

As part of a sample’s overall likelihood, we compute the empirical joint distribution of ligand and pocket nodes *p*(*N*_L_, *N*_P_) observed in the training set and smooth it with a Gaussian filter (*σ* = 1). In the conditional generation scenario, we derive the distribution *p*(*N*_L_∣*N*_P_) and use it for likelihood computations.

For sampling, we can either fix molecule sizes manually or sample the number of ligand nodes from the same distribution given the number of nodes in the target pocket:

13 $${N}_{{\mathrm{L}}} \sim p({N}_{{\mathrm{L}}}| {N}_{{\mathrm{P}}}).$$

For the experiments discussed in ‘DiffSBDD captures the underlying data distribution’ section, we increase the mean size of sampled molecules by five (CrossDocked) and ten (Binding MOAD) atoms, respectively, to approximately match the sizes of molecules found in the test set. This modification makes the reported Vina scores more comparable as the in silico docking score is highly correlated with the molecular size, which is demonstrated in Supplementary Fig. 4. Average molecule sizes after applying the correction are shown in Supplementary Table 7 together with corresponding values for generated molecules from other methods.

#### Featurization

All molecules are expressed as graphs in which every atom is represented by a node. To process ligand and pocket nodes with a single GNN, atom types and residue types are first embedded in a joint node embedding space by separate learnable MLPs (Fig. 1f). We also experimented with coarse-grained *C*_*α*_ descriptions of the pockets to reduce processing time but found this representation to be inferior in most cases (Supplementary Section 5.9). The full atom model uses the same one-hot encoding of atom types for ligand and protein nodes. For the *C*_*α*_-only model, the node features of the protein are set as one-hot encodings of the amino acid type instead.

#### Noise schedule

We use the pre-defined polynomial noise schedule introduced in ref. ^24^:

14 $${\tilde{\alpha }}_{t}=1-{\left(\frac{t}{T}\right)}^{2},\quad t=0,\ldots ,T.$$

Following refs. ^24,43^, values of ${\tilde{\alpha }}_{t| s}^{2}={\left(\frac{{\tilde{\alpha }}_{t}}{{\tilde{\alpha }}_{s}}\right)}^{2}$ are clipped between 0.001 and 1 for numerical stability near *t* = *T*, and ${\tilde{\alpha }}_{t}$ is recomputed as

15 $${\tilde{\alpha }}_{t}=\mathop{\prod }\limits_{\tau =0}^{t}{\tilde{\alpha }}_{\tau | \tau -1}.$$

A tiny offset *ϵ* = 10^−5^ is used to avoid numerical problems at *t* = 0 defining the final noise schedule:

16 $${\alpha }_{t}^{2}=(1-2\epsilon )\cdot {\tilde{\alpha }}_{t}^{2}+\epsilon .$$

#### Feature scaling

We scale the node-type features **h** by a factor of 0.25 relative to the coordinates **x**, which was empirically found to improve model performance in previous work^24^. To train joint probability models in the all-atom scenario, it was necessary to scale down the coordinates (and corresponding distance cut-offs) by a factor of 0.2 instead to avoid introducing too many edges in the graph near the end of the diffusion process at *t* = *T*.

#### Postprocessing

For postprocessing of generated molecules, we use a similar procedure as in ref. ^44^. Given a list of atom types and coordinates, bonds are first added using OpenBabel^45^. We then use RDKit to sanitize molecules and filter for the largest molecular fragment.

#### Quantitative evaluation of inpainting for the whole Binding MOAD test set

For all inpainting experiments across the whole test set, we perform automatic masking of atoms that are to be fixed. For scaffold elaboration, we extract the Bemis–Murcko scaffold^46^ using RDKit and compute a binary mask to fix the scaffold, while functional groups are redesigned. For scaffold hopping, we simply take the inverse of the mask used for scaffold elaboration. For linker design, we fragment each molecule in the test set in multiple ways as in Igashov et al.^36^. To benchmark against DiffLinker, we use the model weights and protocol as described in Igashov et al.^36^ except we give the ground-truth linker size as input, rather than predict it using the auxiliary model, for fairness. In small-scale experiments where finer control is desirable (for example, as in the fragment merging example described below), the binary mask is defined manually.

Depending on the use case, we find it desirable to perform molecular inpainting within two regimes: (1) designing a completely new inpainted region de novo (DiffSBDD-de novo) to explore the entire chemical fitness landscape; or (2) redesigning an existing region via partial noising then denoising (Supplementary Section 5.7), thus locally exploring desired properties by exploitation (DiffSBDD-diversify). The first case is more amenable to situations in which we have no prior information other than the fixed substructure (for example, fragment linking after a fragment screen), meaning that unconstrained exploration of the chemical fitness landscape is the preferred approach for the majority of SBDD. The second case is more relevant in scenarios where we have prior information about the desired chemical and topological composition of the designed region that we can use to bias generation (with the choice of *t* being a hyperparameter). This is particularly relevant in the case of scaffold hopping, where we try to keep the properties of a molecule relatively unchanged while designing a new topology^47^.

#### Molecular-inpainting case studies

All molecular-inpainting experiments shown in Extended Data Fig. 1a–e use a version of DiffSBDD-cond trained on Binding MOAD.

Scaffold hopping is performed for a mitotic kinesin Eg5 inhibitor (PDB code 2gm1)^48^ where we fix the functional groups mediating the binding to the pocket while designing a new scaffold structure.

The opposite case of scaffold elaboration is applied to a rationally designed inhibitor targeting the actin-associated protein ENAH EVH1 (PDB code 6rcj)^49^ where we fix the scaffold and design new functional groups.

Fragment merging is the task of combining fragments with an overlapping binding site^50^. For the example in this study, we replicate the results of Gahbauer et al.^51^, who performed fragment merging of two fragments (PDB codes 5rsw and 5rue) identified by experimental screening^52^ for the SARS-CoV-2 non-structural protein 3 (Nsp3) using the chemoinformatics-based method Fragmenstein^53^. To perform the fragment merge, instead of masking out and reinserting atoms, we instead choose to fix all atoms during generation except the atom on each fragment closest to the other. We need to perform *t* = 200 steps of the DiffSBDD-diversify procedure to allow the model to arrange the atom positions as well as change the atom types. All PDB files were already structurally aligned.

Fragment growing is performed around the central motif of another inhibitor for the ENAH EVH1 target (PDB entry 5ndu)^49^.

The fragment linking example is based on the same target (PDB entry 5ndu). Here we are designing not only a small linker made of a few atoms but rather an entirely new fragment with two connecting linkers to join two outer fragments of the reference ligand.

#### Iterative molecule optimization

To perform property optimization as shown in Fig. 1d, we first noise a molecule from an experimental protein–ligand complex for *t* steps, where *t* ≪ *T*, using the forward diffusion process. From this partially noised sample, we can then denoise the appropriate number of steps with the reverse process until *t* = 0. The stochasticity in this quick noise/denoise process allows us to sample new and diverse candidates of various properties while staying in the same region of chemical space, assuming *t* is small (Supplementary Fig. 3). Note that this approach, which is inspired by Luo et al.^54^, does not allow for direct optimization of specific properties. Instead, it can be regarded as an exploration around the local chemical space while maintaining high shape and chemical complementary via the conditional denoising model.

We extend this idea by combining the partial noising/denoising procedure with a simple evolutionary algorithm that optimizes for specific molecular properties (Fig. 1d). At every stage in the optimization process, we generate 100 new molecules (from either the previous generation or the original molecule in the first case). Molecules are modified via partial noising/denoising with a randomly chosen *t* between 10 and 150. The new molecules are then passed to an oracle/score function (for instance, a docking program or synthetic accessibility predictor) to be ranked. The top-*k* molecules are then selected to seed the new population. In our study, we use *k* = 10.

For the selective kinase design experiment, we additionally pruned any candidates that regress with regard to the on- and off-target docking scores of the original molecule before selecting the top molecules (that is those above or left of the red star in Extended Data Fig. 2f) to bias the molecules to have high affinity to the on-target kinase as well as specificity. The starting molecule has ChEMBL identifier CHEMBL388978.

### Experimental set-up

#### Datasets

We use the CrossDocked dataset^55^ with 100,000 high-quality protein–ligand pairs for training and 100 proteins for testing, following the sequence-based data split of previous studies^15,44^.

We also evaluate our method on a curated dataset of experimentally determined complexed protein–ligand structures from Binding MOAD^28^. We keep pockets with moderately ‘drug-like’ ligands with QED score >0.3 that pass the database’s validity criteria (http://www.bindingmoad.org/). We further discard small molecules that contain atom types ∉ {C, N, O, S, B, Br, Cl, P, I, F} as well as binding pockets with non-standard amino acids. We define binding pockets as the set of residues that have any atom within 8 Å of any ligand atom. Ligand redundancy is reduced by randomly sampling at most 50 molecules with the same chemical component identifier (3-letter-code). After removing corrupted entries that could not be processed, 40,344 training pairs and 130 testing pairs remain. A validation set of size 246 is used to monitor estimated log-likelihoods during training. The split is made to ensure different sets do not contain proteins from the same Enzyme Commission Number main class.

As various proteins could not be successfully processed by one or several baseline methods, our analysis of the distribution learning capabilities is performed for only pockets for which samples from all methods are available. These are 78 and 119 targets from CrossDocked and Binding MOAD, respectively.

#### Baselines

We select four recently published autoregressive deep learning methods for SBDD. Pocket2Mol^15^, ResGen^25^ and PocketFlow^26^ are sequential schemes relying on graph representations of the protein pocket and previously placed atoms to predict probabilities based on which new atoms are added. DeepICL^27^ pursues a similar sequential approach but strives to improve generalizability in the face of limited data by incorporating prior knowledge in the form of protein–ligand interaction patterns. They are currently the state of the art among this class of models. For Pocket2Mol, we re-evaluate already generated ligands on the CrossDocked dataset kindly provided by the authors. All other results were produced using the official implementations available online with default sampling parameters. Note that, unlike DiffSBDD, we therefore sample for the Binding MOAD test set with Pocket2Mol and ResGen models that have been trained on CrossDocked. As these two sets overlap (30 test set proteins from Binding MOAD are found in the CrossDocked training set), there is potential data leakage. In practice, however, we do not observe substantially different results when these targets are excluded from the analysis. We also attempted to train Pocket2Mol on Binding MOAD, but did not manage to robustly train the model on this dataset due to instability during training. PocketFlow was pretrained on about 8 million molecules from the ZINC database^56^ and finetuned on a different subset of the CrossDocked dataset. DeepICL was trained on a much smaller dataset with about 11,000 structures from the PDBbind database^57^.

For the fragment linking task, we compare against DiffLinker^36^. DiffLinker is an equivariant diffusion model similar to ours, but takes the pocket and fixed fragments as inputs and then designs only a linker.

#### Evaluation metrics

We use widely used metrics to assess the quality of our generated molecules^14,15^. (1) Vina score is an empirical estimate of the binding free energy of protein–small-molecule complexes. While it is not an ideal predictor of binding affinity, we chose the Vina score as a fast proxy that shows a certain level of correlation with experimentally determined values (see Extended Data Fig. 3 in ref. ^36^). (2) Convolutional neural network affinity is another predicted affinity score reported by the GNINA docking software^58^. (3) QED is a quantitative estimation of drug-likeness combining several desirable molecular properties^59^. (4) SA estimates synthetic accessibility, that is, the difficulty of synthesis^60^. (5) log*P* is the predicted octanol–water partition coefficient, a measure of hydrophobicity^61^. (6) Lipinski measures how many rules in the Lipinski rule of five^62^ are satisfied (in addition to the original four rules we require ten or fewer rotatable bonds). (7) Diversity is computed as the average pairwise dissimilarity (1 − Tanimoto similarity) between molecular fingerprints of all generated molecules for each pocket. (8) Inference time is the average sampling time per target. Chemical properties are calculated with RDKit^63^. Docking scores are obtained after local minimization with an empirical force field using the GNINA implementation^58^ or, if specified, after redocking with QuickVina2^64^.

#### Statistics and reproducibility

No statistical method was used to predetermine sample size. While we aimed to sample 100 ligands per pocket for the results in the ‘DiffSBDD captures the underlying data distribution’ section, the exact number of available molecules varies slightly due to technical reasons and the characteristics of the different methods (Supplementary Table 9). Some metrics could be calculated only for molecules that pass RDKit’s sanitization step. Molecules not passing this filter were therefore excluded from the affected analyses. Furthermore, we exclude DeepICL from the comparison with Binding MOAD as we did not manage to sample any molecules for more than half of the test set proteins. Nevertheless we report distribution learning results of all methods on this substantially reduced set of targets in Supplementary Section 5.2.

#### Software

All code was written in Python (v3.10.4). For dataset preparation, we used numpy (v1.22.4), BioPython (v1.81) and RDKit (v2023.9.4). The neural network models were implemented and trained with PyTorch (v1.12.1), PyTorch Lightning (v1.7.4), PyTorch Geometric (v2.2) and Weights & Biases (v0.13.1). OpenBabel (v3.1.1) and RDKit (v2023.9.4) were used to post-process molecules. Docking/scoring was performed using the Gnina (v1.1) and QuickVina (v2.1) softwares. The data were analyzed and visualized using Pandas (v1.4.2), SciPy (v1.7.3), Matplotlib (v3.4.3) and Seaborn (v0.12.0).

The code required to run the baseline models is available in public repositories. Pocket2Mol can be found at https://github.com/pengxingang/Pocket2Mol, ResGen at https://github.com/HaotianZhangAI4Science/ResGen, PocketFlow (latest) at https://github.com/Saoge123/PocketFlow, and DeepICL (v1.1.0) at https://github.com/ACE-KAIST/DeepICL. Finally, DiffLinker (v1.0) is available at https://github.com/igashov/DiffLinker. The Pocket2Mol and ResGen repositories do not provide version releases.

### Reporting summary

Further information on research design is available in the Nature Portfolio Reporting Summary linked to this article.

## Supplementary information

Supplementary Information Supplementary Figs. 1–9, Tables 1–11, Algorithm 1, proofs, extended results and discussions, and additional references.

Reporting Summary

## Source data

Source Data Fig. 2 Source data for Fig. 2a,b,d,e.

Source Data Extended Data Fig. 1 Source data for Extended Data Fig. 1g.

Source Data Extended Data Fig. 2 Source data for Extended Data Fig. 2b–d,f.

## Data Availability

The subset of the CrossDocked dataset used in this study was curated in a previous work and is available online https://github.com/pengxingang/Pocket2Mol/tree/main/data. The raw Binding MOAD data can be downloaded from http://www.bindingmoad.org/. We provide further instructions on how to process these data in our code repository at https://github.com/arneschneuing/DiffSBDD. Pre-processed versions of both datasets^65^ as well as sampled molecules^66^ are available on Zenodo. Structural models of the discussed protein targets are available under PDB accession codes 2buj (ref. ^67^), 2gm1 (ref. ^68^), 4tos (ref. ^69^), 4w9w (ref. ^70^), 5ndu (ref. ^71^), 5rsw (ref. ^72^), 5rue (ref. ^73^), 5spd (ref. ^74^), 6c0b (ref. ^75^) and 6rcj (ref. ^76^). The starting molecule from the selective kinase design experiment has ChEMBL identifier CHEMBL388978. Source data are provided with this paper.

## Code availability

Our source codes are publicly available at https://github.com/arneschneuing/DiffSBDD (ref. ^77^). Model weights can be downloaded from Zenodo at 10.5281/zenodo.8183747 (ref. ^78^).
