# Supplementary material for: Structure-based drug design with equivariant diffusion models
Source: Nat Comput Sci. 2024 Dec 9;4(12):899–909. doi: 10.1038/s43588-024-00737-x (PMC11659159; doi:10.1038/s43588-024-00737-x)
Supplement: Supplementary file 1 — Supplementary Figs. 1–9, Tables 1–11, Algorithm 1, proofs, extended results and discussions, and additional references. [file 43588_2024_737_MOESM1_ESM.pdf]

nature computational science

Supplementary information https://doi.org/10.1038/s43588-024-00737-x

Structure-based drug design with equivariant diffusion models

In the format provided by the authors and unedited

Supplementary Information for “Structure-based Drug Design with Equivariant Diffusion Models”

Contents

1 Variational Lower Bound **2**

2 Note on Equivariance of the Conditional Model **2**

3 Proofs **2**
3.1 $O(3)$-equivariance of the prior probability . . . 3
3.2 $O(3)$-equivariance of the transition probabilities . . . 3
3.3 $O(3)$-equivariance of the learned likelihood . . . 4

4 $SE(3)$-equivariant Graph Neural Network **4**
4.1 Discussion of equivariance . . . 4
4.2 Empirical results . . . 5

5 Extended Results **6**
5.1 Distribution learning performance . . . 6
5.2 Binding MOAD analysis on a reduced test set . . . 7
5.3 Additional molecular metrics . . . 8
5.4 Selecting the number of resampling steps for DiffSBDD-joint . . . 9
5.5 Quantitative performance of substructure-constrained models . . . 11
5.6 Importance of resampling for molecular substructure inpainting . . . 12
5.7 Diversification of candidate molecules . . . 13
5.8 Dependence of Vina scores on molecule size . . . 14
5.9 Results with a coarse-grained pocket representation . . . 15

6 Supplementary Tables **17**
6.1 Sampling statistics for the distribution learning benchmark . . . 17
6.2 Sampling statistics for the substructure design experiment . . . 18
6.3 Training hyperparameters . . . 19
6.4 Replacement method algorithm . . . 20

7 Supplementary Figures **21**
7.1 Distributions of molecular properties . . . 21
7.2 Generated molecules . . . 23

8 Related Work **25**

References **26**

Supplementary Section 1: Variational Lower Bound

To maximise the likelihood of our training data, we aim at optimising the variational lower bound (VLB) [1, 2]

$$-\log p(\boldsymbol{z}_{\text{data}}) \leq \underbrace{D_{\text{KL}}\big(q(\boldsymbol{z}_T|\boldsymbol{z}_{\text{data}})||p(\boldsymbol{z}_T)\big)}_{\text{prior loss } \mathcal{L}_{\text{prior}}} \underbrace{-\mathbb{E}_{q(\boldsymbol{z}_0|\boldsymbol{z}_{\text{data}})}\big[\log p(\boldsymbol{z}_{\text{data}}|\boldsymbol{z}_0)\big]}_{\text{reconstruction loss } \mathcal{L}_0} + \underbrace{\sum_{t=1}^{T} \mathcal{L}_t}_{\text{diffusion loss}} \tag{1}$$

with

$$\mathcal{L}_t = D_{\text{KL}}\big(q(\boldsymbol{z}_{t-1}|\boldsymbol{z}_{\text{data}}, \boldsymbol{z}_t)||p_\theta(\boldsymbol{z}_{t-1}|\hat{\boldsymbol{z}}_{\text{data}}, \boldsymbol{z}_t)\big) \tag{2}$$

$$= \mathbb{E}_{\boldsymbol{\epsilon}\sim\mathcal{N}(\boldsymbol{0},\boldsymbol{I})}\Big[\frac{1}{2}\Big(\frac{\text{SNR}(t-1)}{\text{SNR}(t)} - 1\Big)||\boldsymbol{\epsilon} - \hat{\boldsymbol{\epsilon}}_\theta||^2\Big] \tag{3}$$

during training. Here, $\boldsymbol{z}_{\text{data}}$ is a training data point, $\boldsymbol{z}_t$ a noised version of that data at time step t and $\hat{\boldsymbol{z}}_{\text{data}}$ an estimate of the clean data based on the noisy data. The subscript θ denotes that a function is parameterized with learnable parameters θ. $\hat{\boldsymbol{\epsilon}}_\theta$ is the neural network output which approximates the noise sample used to perturb the original data point. Note the prior loss should always be close to zero and can be computed exactly in closed form while the reconstruction loss must be estimated as described in Hoogeboom et al. [2]. In practice, however, we simply minimise the mean squared error $\mathcal{L}_{\text{train}} = \frac{1}{2}||\boldsymbol{\epsilon} - \hat{\boldsymbol{\epsilon}}||^2$ while randomly sampling time steps $t \sim \mathcal{U}(0, \ldots, T)$, which is equivalent up to a multiplicative factor.

Supplementary Section 2: Note on Equivariance of the Conditional Model

The 3D-conditional model (DiffSBDD-cond) can achieve equivariance without the usual "subspace-trick". The coordinates of pocket nodes provide a reference frame for all samples that can be used to translate them to a unique location (for instance such that the pocket is centered at the origin: $\sum_i \boldsymbol{x}_i^{(P)} = \boldsymbol{0}$). By doing this for all training data, translation equivariance becomes irrelevant and the CoM-free subspace approach obsolete. To evaluate the likelihood of translated samples at inference time, we can first subtract the pocket's center of mass from the whole system and compute the likelihood after this mapping. Similarly, for sampling molecules we can first generate a ligand in a CoM-free version of the pocket and move the whole system back to the original location of the pocket nodes to restore translation equivariance. As long as the mean of our Gaussian noise distribution depends equivariantly on the pocket node coordinates $\boldsymbol{x}^{(P)}$, $O(3)$-equivariance is satisfied as well (Supplementary Section 3). Since this change did not seem to affect the performance of the conditional model in our experiments, we decided to keep sampling in the linear subspace to ensure that the implementation is as similar as possible to the DiffSBDD-joint model, for which the subspace approach is necessary.

Supplementary Section 3: Proofs

In the following proofs we do not consider categorical node features $\boldsymbol{h}$ as only the positions $\boldsymbol{x}$ are subject to equivariance constraints. Furthermore, we do not distinguish between the zeroth latent representation $\boldsymbol{x}_0$ and data domain representations $\boldsymbol{x}_{\text{data}}$ for ease of notation, and simply drop the subscripts and use $\boldsymbol{x} \in \mathbb{R}^3$.

Supplementary Section 3.1: $O(3)$**-equivariance of the prior probability**

The isotropic Gaussian prior $p(\boldsymbol{x}_T^{(L)}|\boldsymbol{x}^{(P)}) = \mathcal{N}(\boldsymbol{\mu}(\boldsymbol{x}^{(P)}), \sigma^2\boldsymbol{I})$ is equivariant to rotations and reflections represented by an orthogonal matrix $\boldsymbol{R} \in \mathbb{R}^{3\times 3}$ as long as $\boldsymbol{\mu}(\boldsymbol{R}\boldsymbol{x}^{(P)}) = \boldsymbol{R}\boldsymbol{\mu}(\boldsymbol{x}^{(P)})$ because:

$$
\begin{aligned}
p(\boldsymbol{R}\boldsymbol{x}_T^{(L)}|\boldsymbol{R}\boldsymbol{x}^{(P)}) &= \frac{1}{\sqrt{(2\pi)^3}\sigma^3}\exp\Big(-\frac{1}{2\sigma^2}||\boldsymbol{R}\boldsymbol{x}_T^{(L)} - \boldsymbol{\mu}(\boldsymbol{R}\boldsymbol{x}^{(P)})||^2\Big) \\
&= \frac{1}{\sqrt{(2\pi)^3}\sigma^3}\exp\Big(-\frac{1}{2\sigma^2}||\boldsymbol{R}\boldsymbol{x}_T^{(L)} - \boldsymbol{R}\boldsymbol{\mu}(\boldsymbol{x}^{(P)})||^2\Big) \\
&= \frac{1}{\sqrt{(2\pi)^3}\sigma^3}\exp\Big(-\frac{1}{2\sigma^2}||\boldsymbol{R}(\boldsymbol{x}_T^{(L)} - \boldsymbol{\mu}(\boldsymbol{x}^{(P)}))||^2\Big) \\
&= \frac{1}{\sqrt{(2\pi)^3}\sigma^3}\exp\Big(-\frac{1}{2\sigma^2}||\boldsymbol{x}_T^{(L)} - \boldsymbol{\mu}(\boldsymbol{x}^{(P)})||^2\Big) \\
&= p(\boldsymbol{x}_T^{(L)}|\boldsymbol{x}^{(P)}).
\end{aligned}
$$

Here we used $||\boldsymbol{R}\boldsymbol{x}||_2 = ||\boldsymbol{x}||_2$ for orthogonal $\boldsymbol{R}$.

Supplementary Section 3.2: $O(3)$**-equivariance of the transition probabilities**

The denoising transition probabilities from time step t to $s < t$ are defined as isotropic normal distributions:

$$
p_\theta(\boldsymbol{x}_{t-1}^{(L)}|\boldsymbol{x}_t^{(L)}, \hat{\boldsymbol{x}}^{(L)}, \boldsymbol{x}^{(P)}) = \mathcal{N}(\boldsymbol{x}_{t-1}^{(L)}|\boldsymbol{\mu}_{t\to s}(\boldsymbol{x}_t^{(L)}, \hat{\boldsymbol{x}}^{(L)}, \boldsymbol{x}^{(P)}), \sigma_{t\to s}^2\boldsymbol{I}). \tag{4}
$$

Therefore, $p_\theta(\boldsymbol{x}_{t-1}^{(L)}|\boldsymbol{x}_t^{(L)}, \hat{\boldsymbol{x}}^{(L)}, \boldsymbol{x}^{(P)})$ is $O(3)$-equivariant by a similar argument to Supplementary Section 3.1 if $\boldsymbol{\mu}_{t\to s}$ is computed equivariantly from the three-dimensional context.

Recalling the definition of $\boldsymbol{\mu}_{t\to s} = \frac{\alpha_{t|s}\sigma_s^2}{\sigma_t^2}\boldsymbol{x}_t^{(L)} + \frac{\alpha_s\sigma_{t|s}^2}{\sigma_t^2}\hat{\boldsymbol{x}}^{(L)}$, we can prove its equivariance as follows:

$$
\begin{aligned}
\boldsymbol{\mu}_{t\to s}(\boldsymbol{R}\boldsymbol{x}_t^{(L)}, \boldsymbol{R}\boldsymbol{x}^{(P)}) &= \frac{\alpha_{t|s}\sigma_s^2}{\sigma_t^2}\boldsymbol{R}\boldsymbol{x}_t^{(L)} + \frac{\alpha_s\sigma_{t|s}^2}{\sigma_t^2}\hat{\boldsymbol{x}}^{(L)}(\boldsymbol{R}\boldsymbol{x}_t^{(L)}, \boldsymbol{R}\boldsymbol{x}^{(P)}) \\
&= \frac{\alpha_{t|s}\sigma_s^2}{\sigma_t^2}\boldsymbol{R}\boldsymbol{x}_t^{(L)} + \frac{\alpha_s\sigma_{t|s}^2}{\sigma_t^2}\boldsymbol{R}\hat{\boldsymbol{x}}^{(L)}(\boldsymbol{x}_t^{(L)}, \boldsymbol{x}^{(P)}) \quad \text{(equivariance of } \hat{\boldsymbol{x}}^{(L)}\text{)} \\
&= \boldsymbol{R}\Big(\frac{\alpha_{t|s}\sigma_s^2}{\sigma_t^2}\boldsymbol{x}_t^{(L)} + \frac{\alpha_s\sigma_{t|s}^2}{\sigma_t^2}\hat{\boldsymbol{x}}^{(L)}(\boldsymbol{x}_t^{(L)}, \boldsymbol{x}^{(P)})\Big) \\
&= \boldsymbol{R}\boldsymbol{\mu}_{t\to s}(\boldsymbol{x}_t^{(L)}, \boldsymbol{x}^{(P)}),
\end{aligned}
$$

where $\hat{\boldsymbol{x}}^{(L)}$, defined as $\hat{\boldsymbol{x}}^{(L)} = \frac{1}{\alpha_t}\boldsymbol{x}_t^{(L)} - \frac{\sigma_t}{\alpha_t}\hat{\boldsymbol{\epsilon}}$, is equivariant because:

$$
\begin{aligned}
\hat{\boldsymbol{x}}^{(L)}(\boldsymbol{R}\boldsymbol{x}_t^{(L)}, \boldsymbol{R}\boldsymbol{x}^{(P)}) &= \frac{1}{\alpha_t}\boldsymbol{R}\boldsymbol{x}_t^{(L)} - \frac{\sigma_t}{\alpha_t}\hat{\boldsymbol{\epsilon}}(\boldsymbol{R}\boldsymbol{x}_t^{(L)}, \boldsymbol{R}\boldsymbol{x}^{(P)}, t) \\
&= \frac{1}{\alpha_t}\boldsymbol{R}\boldsymbol{x}_t^{(L)} - \frac{\sigma_t}{\alpha_t}\boldsymbol{R}\hat{\boldsymbol{\epsilon}}(\boldsymbol{x}_t^{(L)}, \boldsymbol{x}^{(P)}, t) \quad (\hat{\boldsymbol{\epsilon}} \text{ predicted by equivariant neural network}) \\
&= \boldsymbol{R}\Big(\frac{1}{\alpha_t}\boldsymbol{x}_t^{(L)} - \frac{\sigma_t}{\alpha_t}\hat{\boldsymbol{\epsilon}}(\boldsymbol{x}_t^{(L)}, \boldsymbol{x}^{(P)}, t)\Big) \\
&= \boldsymbol{R}\hat{\boldsymbol{x}}^{(L)}(\boldsymbol{x}_t^{(L)}, \boldsymbol{x}^{(P)}).
\end{aligned}
$$

Supplementary Section 3.3: $O(3)$-equivariance of the learned likelihood

Let $\boldsymbol{R} \in \mathbb{R}^{3\times 3}$ be an orthogonal matrix representing an element g from the general orthogonal group $O(3)$. We obtain the marginal probability density of the Markovian denoising process as follows

$$\begin{aligned} p_\theta(\boldsymbol{x}_0^{(L)}|\boldsymbol{x}^{(P)}) &= \int p(\boldsymbol{x}_T^{(L)}|\boldsymbol{x}^{(P)}) p_\theta(\boldsymbol{x}_{0:T-1}^{(L)}|\boldsymbol{x}_T^{(L)}, \boldsymbol{x}^{(P)}) \mathrm{d}\boldsymbol{x}_{1:T} \\ &= \int p(\boldsymbol{x}_T^{(L)}|\boldsymbol{x}^{(P)}) \prod_{t=1}^{T} p_\theta(\boldsymbol{x}_{t-1}^{(L)}|\boldsymbol{x}_t^{(L)}, \boldsymbol{x}^{(P)}) \mathrm{d}\boldsymbol{x}_{1:T} \end{aligned}$$

and the sample's likelihood is $O(3)$-equivariant:

$$\begin{aligned} p_\theta(\boldsymbol{R}\boldsymbol{x}_0^{(L)}|\boldsymbol{R}\boldsymbol{x}^{(P)}) &= \int p(\boldsymbol{R}\boldsymbol{x}_T^{(L)}|\boldsymbol{R}\boldsymbol{x}^{(P)}) \prod_{t=1}^{T} p_\theta(\boldsymbol{R}\boldsymbol{x}_{t-1}^{(L)}|\boldsymbol{R}\boldsymbol{x}_t^{(L)}, \boldsymbol{R}\boldsymbol{x}^{(P)}) \mathrm{d}\boldsymbol{x}_{1:T} \\ &= \int p(\boldsymbol{x}_T^{(L)}|\boldsymbol{x}^{(P)}) \prod_{t=1}^{T} p_\theta(\boldsymbol{R}\boldsymbol{x}_{t-1}^{(L)}|\boldsymbol{R}\boldsymbol{x}_t^{(L)}, \boldsymbol{R}\boldsymbol{x}^{(P)}) \mathrm{d}\boldsymbol{x}_{1:T} \quad \text{(equivariant prior)} \\ &= \int p(\boldsymbol{x}_T^{(L)}|\boldsymbol{x}^{(P)}) \prod_{t=1}^{T} p_\theta(\boldsymbol{x}_{t-1}^{(L)}|\boldsymbol{x}_t^{(L)}, \boldsymbol{x}^{(P)}) \mathrm{d}\boldsymbol{x}_{1:T} \quad \text{(equivariant transition probabilities)} \\ &= p_\theta(\boldsymbol{x}_0^{(L)}|\boldsymbol{x}^{(P)}). \end{aligned}$$

Supplementary Section 4: $SE(3)$-equivariant Graph Neural Network

Chiral molecules cannot be superimposed by any combination of rotations and translations. Instead they are mirrored along a stereocenter, axis, or plane. As chirality can fundamentally alter a molecule's chemical properties, we use a variant of the $E(3)$-equivariant graph neural networks [3] that is sensitive to reflections and hence $SE(3)$-equivariant. We change the coordinate update equation of standard EGNNs in the following way

$$\boldsymbol{x}_i^{l+1} = \boldsymbol{x}_i^l + \sum_{j\neq i} \frac{\boldsymbol{x}_i^l - \boldsymbol{x}_j^l}{d_{ij}+1} \phi_x^d(\boldsymbol{h}_i^l, \boldsymbol{h}_j^l, d_{ij}^2, a_{ij}) + \frac{(\boldsymbol{x}_i^l - \bar{\boldsymbol{x}}^l) \times (\boldsymbol{x}_j^l - \bar{\boldsymbol{x}}^l)}{||(\boldsymbol{x}_i^l - \bar{\boldsymbol{x}}^l) \times (\boldsymbol{x}_j^l - \bar{\boldsymbol{x}}^l)|| + 1} \phi_x^\times(\boldsymbol{h}_i^l, \boldsymbol{h}_j^l, d_{ij}^2, a_{ij}), \quad (5)$$

where $\bar{\boldsymbol{x}}^l$ denotes the center of mass of all nodes at layer l. This modification makes the EGNN layer sensitive to reflections while staying close to the original formalism. Since the resulting graph neural networks are only equivariant to the $SE(3)$ group, we will hereafter call them SE(3)GNNs for short.

Supplementary Section 4.1: Discussion of equivariance

Here we study how the suggested change in the coordinate update equation breaks reflection symmetry while preserving equivariance to rotations. Messages and scalar feature updates (Equations 3 and 4 in the Methods Section) remain $E(3)$-invariant as in the original model and are therefore not considered in this section. We analyze transformations composed of a translation by $\boldsymbol{t} \in \mathbb{R}^3$ and a rotation/reflection by an orthogonal matrix $\boldsymbol{R} \in \mathbb{R}^{3\times 3}$ with $\boldsymbol{R}^T\boldsymbol{R} = \boldsymbol{I}$. The output at layer $l+1$ given the transformed input $\boldsymbol{R}\boldsymbol{x}_i^l + \boldsymbol{t}$ at layer l is calculated as:

$$\boldsymbol{R}\boldsymbol{x}_i^l + \boldsymbol{t} + \sum_{j\neq i} \frac{\boldsymbol{R}\boldsymbol{x}_i^l + \boldsymbol{t} - (\boldsymbol{R}\boldsymbol{x}_j^l + \boldsymbol{t})}{d_{ij}+1} \phi_x^d(\cdot) + \frac{(\boldsymbol{R}\boldsymbol{x}_i^l + \boldsymbol{t} - (\boldsymbol{R}\bar{\boldsymbol{x}}^l + \boldsymbol{t})) \times (\boldsymbol{R}\boldsymbol{x}_j^l + \boldsymbol{t} - (\boldsymbol{R}\bar{\boldsymbol{x}}^l + \boldsymbol{t}))}{Z_{ij}^\times + 1} \phi_x^\times(\cdot) \quad (6)$$

$$= \boldsymbol{R}\boldsymbol{x}_i^l + \boldsymbol{t} + \sum_{j\neq i} \frac{\boldsymbol{R}(\boldsymbol{x}_i^l - \boldsymbol{x}_j^l)}{d_{ij}+1} \phi_x^d(\cdot) + \frac{(\boldsymbol{R}\boldsymbol{x}_i^l - \boldsymbol{R}\bar{\boldsymbol{x}}^l) \times (\boldsymbol{R}\boldsymbol{x}_j^l - \boldsymbol{R}\bar{\boldsymbol{x}}^l)}{Z_{ij}^\times + 1} \phi_x^\times(\cdot) \quad (7)$$

$$= \boldsymbol{R}\boldsymbol{x}_i^l + \boldsymbol{t} + \sum_{j \neq i} \frac{\boldsymbol{R}(\boldsymbol{x}_i^l - \boldsymbol{x}_j^l)}{d_{ij} + 1} \phi_x^d(\cdot) + \frac{\det(\boldsymbol{R})\boldsymbol{R}\big((\boldsymbol{x}_i^l - \bar{\boldsymbol{x}}^l) \times (\boldsymbol{x}_j^l - \bar{\boldsymbol{x}}^l)\big)}{Z_{ij}^{\times} + 1} \phi_x^{\times}(\cdot) \tag{8}$$

$$= \boldsymbol{R}\boldsymbol{x}_i^{l+1} + \boldsymbol{t} + \big(\det(\boldsymbol{R}) - 1\big) \sum_{j \neq i} \frac{\boldsymbol{R}\big((\boldsymbol{x}_i^l - \bar{\boldsymbol{x}}^l) \times (\boldsymbol{x}_j^l - \bar{\boldsymbol{x}}^l)\big)}{Z_{ij}^{\times} + 1}. \tag{9}$$

This result shows that the output coordinates are only equivariantly transformed if $\boldsymbol{R}$ is orientation preserving, that is $\det(\boldsymbol{R}) = 1$. If $\boldsymbol{R}$ is a reflection ($\det(\boldsymbol{R}) = -1$), coordinates will be updated with an additional summand that breaks the symmetry.

The learnable coefficients $\phi_x^d(\boldsymbol{h}_i^l, \boldsymbol{h}_j^l, d_{ij}^2, a_{ij})$ and $\phi_x^{\times}(\boldsymbol{h}_i^l, \boldsymbol{h}_j^l, d_{ij}^2, a_{ij})$ only depend on relative distances and are therefore $E(3)$-invariant. Their arguments are represented with the "$\cdot$" symbol for brevity. Likewise, the normalization factor $||(\boldsymbol{x}_i^l - \bar{\boldsymbol{x}}^l) \times (\boldsymbol{x}_j^l - \bar{\boldsymbol{x}}^l)||$ is abbreviated as $Z_{ij}^{\times}$. Already in the first line we used the fact that the mean transforms equivariantly. Furthermore, we use $\boldsymbol{R}\boldsymbol{a} \times \boldsymbol{R}\boldsymbol{b} = \det(\boldsymbol{R})\boldsymbol{R}(\boldsymbol{a} \times \boldsymbol{b})$ in the second step, which can be derived as follows:

$$\boldsymbol{x}^T(\boldsymbol{R}\boldsymbol{a} \times \boldsymbol{R}\boldsymbol{b}) = \det(\underbrace{[\boldsymbol{x}, \boldsymbol{R}\boldsymbol{a}, \boldsymbol{R}\boldsymbol{b}]}_{\in \mathbb{R}^{3 \times 3}}) \tag{10}$$

$$= \det(\boldsymbol{R}[\boldsymbol{R}^T\boldsymbol{x}, \boldsymbol{a}, \boldsymbol{b}]) \tag{11}$$

$$= \det(\boldsymbol{R}) \det([\boldsymbol{R}^T\boldsymbol{x}, \boldsymbol{a}, \boldsymbol{b}]) \tag{12}$$

$$= \det(\boldsymbol{R}) \Big(\boldsymbol{x}^T\boldsymbol{R}(\boldsymbol{a} \times \boldsymbol{b})\Big) \tag{13}$$

$$= \boldsymbol{x}^T \Big(\det(\boldsymbol{R})\boldsymbol{R}(\boldsymbol{a} \times \boldsymbol{b})\Big). \tag{14}$$

The stated property of the cross product follows because this derivation is true for all $\boldsymbol{x} \in \mathbb{R}^3$.

Supplementary Section 4.2: Empirical results

To show the effectiveness of this architecture on a simple toy example, we repeat the classification experiment by Adams et al. [4] who train neural networks to classify tetrahedral chiral centers as right-handed (*rectus*, 'R') or left-handed (*sinister*, 'S'). We closely follow their data split and experimental set-up and only replace the classifier with EGNN and SE(3)GNNs, respectively. The results in Supplementary Table 1 clearly demonstrate that the $SE(3)$-equivariant EGNN is capable of solving this task (without any hyperparameter optimization) whereas the $E(3)$-equivariant version does not do better than random guessing.

Supplementary Table 1: Accuracy on the R/S classification task. Results in the first section are taken from [4] and included for reference.

Model	R/S Accuracy (%)
ChIRo	98.5
SchNet	54.4
DimeNet++	65.7
SphereNet	98.2
EGNN	50.4
SE(3)GNN	83.4

Supplementary Section 5: Extended Results

Supplementary Section 5.1: Distribution learning performance

Since distribution learning capabilities in the high-dimensional space of chemical compounds are difficult to quantify directly, we instead measure a range of molecular properties that are relevant for potential drug candidates. We then compare the distributions of these scores to the distributions we get from the real ligands in our test set using the Wasserstein distance. These results are summarized in Supplementary Table 2 for computational scores of drug-likeness (QED), synthetic accessibility (SA), hydrophobicity (LogP) and two measures of target affinity, the empirical Vina scoring function and a neural network estimation of binding affinity (CNN affinity). Both were computed by GNINA [5] after local energy minimization to resolve minor clashes. The underlying distributions of scores are shown in Supplementary Figures 6 and 7. We perform this analysis both for the test set targets from CrossDocked [6], a standard dataset extensively used in prior works [7–10], and our newly curated dataset based on Binding MOAD [11]. Note that not all baseline models have been trained on identical training sets (see Methods Section 2.6). Since we were only able to sample molecules for a fraction of our Binding MOAD test pockets with DeepICL, we decided to exclude it from this comparison. Readers are referred to Supplementary Table 3 for a summary of the results on the reduced test set. Generally, our diffusion models capture molecular properties of natural ligands more accurately than the autoregressive baselines despite substantially shorter sampling times in most cases. A notable exception is the Vina score, which Pocket2Mol matches particularly well on the CrossDocked dataset. Interestingly, this observation is not confirmed by GNINA's CNN affinity which estimates the same quantity. Its distribution is better approximated by DiffSBDD.

Supplementary Table 2: Evaluation of generated molecules for targets from the CrossDocked and Binding MOAD test sets. To assess how well the models capture properties of real ligands, we compute the Wasserstein distance between the distributions of a scores from generated molecules and the ground truth molecules from the test sets. The best performance (lowest Wasserstein distance) is highlighted in bold. * denotes that we re-evaluate the generated ligands provided by the authors. † means inference times are taken from the original paper. ‡ means inference time estimated based on five targets.
QED: Quantitative Estimation of Drug-likeness [12]; *SA*: Synthetic Accessibility [13]; *LogP*: octanol-water partition coefficient [14]; *CNN affinity*: estimated binding affinity score using a Convolutional Neural Network [5].

		Wasserstein distance to reference distribution (↓)						
		QED	SA	LogP	Lipinski	Vina score	CNN affinity	Time (s, ↓)
CrossDocked	Pocket2Mol [9]*	0.104	**0.243**	0.908	0.656	**0.589**	0.608	$2504 \pm 2207^{\dagger}$
	ResGen [10]	0.114	0.554	0.794	0.682	2.13	1.2	$\approx 936^{\ddagger}$
	PocketFlow [15]	0.0617	0.78	2.79	0.648	1.18	0.897	193.5 ± 18.1
	DeepICL [16]	0.147	3.54	1.21	0.666	1.16	0.306	300.5 ± 65.5
	DiffSBDD-cond	**0.0191**	1.29	**0.601**	**0.272**	0.83	**0.146**	$\mathbf{135.9 \pm 51.7}$
	DiffSBDD-joint	0.0193	1.51	1.7	0.416	0.683	0.273	160.3 ± 73.3
Binding MOAD	Pocket2Mol [9]	0.124	0.873	0.823	0.276	**3.3**	1.57	$\approx 613^{\ddagger}$
	ResGen [10]	0.0761	0.878	0.831	0.248	6.75	1.73	$\approx 697^{\ddagger}$
	PocketFlow [15]	**0.0612**	1.09	1.92	0.272	3.41	1.53	$\mathbf{185.79 \pm 17.8}$
	DiffSBDD-cond	0.0904	1.2	**0.801**	**0.142**	4.19	0.902	336.1 ± 85.0
	DiffSBDD-joint	0.0734	**0.795**	1.28	0.19	9.63	**0.627**	369.9 ± 124.5

Supplementary Section 5.2: Binding MOAD analysis on a reduced test set

We were only able to sample molecules with the DeepICL [16] baseline method for less than 50% of pockets in our Binding MOAD test set. We therefore include distribution learning results on this reduced set of pockets in Supplementary Table 3 for completeness.

Supplementary Table 3: Evaluation of generated molecules for 54 targets from the Binding MOAD test set. To assess how well the models capture properties of real ligands, we compute the Wasserstein distance between the distributions of a scores from generated molecules and the ground truth molecules from the test sets. The best performance (lowest Wasserstein distance) is highlighted in bold.
QED: Quantitative Estimation of Drug-likeness [12]; *SA*: Synthetic Accessibility [13]; *LogP*: octanol-water partition coefficient [14]; *CNN affinity*: estimated binding affinity score using a Convolutional Neural Network [5].

	Wasserstein distance to reference distribution (↓)					
	QED	SA	LogP	Lipinski	Vina score	CNN affinity
Pocket2Mol [9]	0.1	0.569	1.06	0.249	**3.57**	1.77
ResGen [10]	**0.0583**	**0.525**	1.01	0.178	8.71	1.88
PocketFlow [15]	0.0751	0.748	0.678	0.212	3.84	1.95
DeepICL [16]	0.0641	3.72	2.34	0.19	3.58	1.27
DiffSBDD-cond	0.166	1.59	1.66	0.168	4.44	1.03
DiffSBDD-joint	0.0812	1.15	**0.619**	**0.13**	7.99	**0.871**

Supplementary Section 5.3: Additional molecular metrics

In addition to the molecular properties discussed in the main text we assess the models' ability to produce novel and valid molecules using four simple metrics: validity, connectivity, uniqueness, and novelty. **Validity** measures the proportion of generated molecules that pass basic tests by RDKit– mostly ensuring correct valencies. **Connectivity** is the proportion of valid molecules that do not contain any disconnected fragments. We convert every valid and connected molecule from a graph into a canonical SMILES string representation, count the number unique occurrences in the set of generated molecules and compare those to the training set SMILES to compute **uniqueness** and **novelty** respectively.

Supplementary Table 4 shows that only a small fraction of all generated molecules is invalid and must be discarded for downstream processing. A much larger percentage of molecules is fragmented but, since we can simply select and process the largest fragments in these cases, low connectivity does not necessarily affect the efficiency of the generative process. Moreover, all models produce diverse sets of molecules unseen in the training set.

Supplementary Table 4: Basic molecular metrics for generated small molecules given a C_α and full atom representation of the protein pocket.

**Model**	**Validity**	**Connectivity**	**Uniqueness**	**Novelty**
CrossDocked test set	100%	100%	96%	96.88%
DiffSBDD-cond (C_α)	95.52%	79.52%	99.99%	99.97%
DiffSBDD-joint (C_α)	99.18%	98.25%	99.52%	99.97%
DiffSBDD-cond	97.10%	78.27%	99.98%	99.99%
DiffSBDD-joint	92.99%	67.52%	100%	100%
Binding MOAD test set	97.69%	100%	38.58%	77.55%
DiffSBDD-cond (C_α)	94.41%	77.38%	100%	100%
DiffSBDD-joint (C_α)	98.36%	91.60%	99.99%	99.98%
DiffSBDD-cond	96.32%	63.37%	100%	100%
DiffSBDD-joint	93.88%	75.60%	100%	100%

Supplementary Section 5.4: Selecting the number of resampling steps for DiffSBDD-joint

Here, we briefly recapitulate the resampling algorithm introduced in Ref. [17]. The key intuition is that inpainting with the replacement method combines a generated part with an independently sampled latent representation of the known part. Even though the neural network tries to reconcile these two components in every step of the denoising trajectory, it cannot succeed because the same issue reoccurs in the following step. Lugmayr et al. [17] thus propose to apply the neural network several times before proceeding to the next noise level, allowing the DDPM to preserve more conditional information and move the sample closer to the data distribution again.

Number of resampling steps

To empirically study the effect of the number of resampling iterations applied, we generated ligands for all test pockets with $r = 1$, $r = 5$, and $r = 10$ resampling steps, respectively. Because the resampling strategy slows down sampling approximately by a factor of r, we used the striding technique proposed by Nichol and Dhariwal [18] and reduced the number of denoising steps proportionally to r. Nichol and Dhariwal [18] showed that this approach reduces the number of sampling steps substantially without sacrificing sample quality. In our case, it allows us to retain sampling speed while increasing the number of resampling steps.

To gauge the effect of resampling for molecule generation we show the distribution of RMSD values between the center of mass of reference molecules and generated molecules in Supplementary Figure 1. The unmodified replacement method ($r = 1$) produces molecules that are clearly farther away from the presumed pocket center than the conditional model. Increasing r moves the mean distance closer to the average displacement of molecules from the conditional method. This effect seems to saturate at $r = 10$ which is in line with the results obtained for images [17].

Supplementary Table 5 shows that neither the additional resampling steps nor the shortened denoising trajectory degrade the performance on the reported molecular metrics. The average docking scores even improve slightly which might reflect better positioning of generated ligands in the pockets prior to docking. The same model trained with $T = 500$ diffusion steps was used in all three cases.

Supplementary Table 5: Evaluation of generated molecules for target pockets from the CrossDocked (C.D.) and Binding MOAD (B.M.) test sets with the inpainting approach and C_α pocket representation for varying numbers of resampling steps r and denoising steps T. Mean and standard deviation are computed across all generated molecules for Vina Score, QED, SA_{norm} and Lipinski. The same statistics are derived for the per-target values of Diversity and Time, respectively. Here, the SA scores were mapped to the unit interval using $\text{SA}_{\text{norm}} = (10 - \text{SA})/9$.

	r	T	Vina Score (↓)	QED (↑)	SA_{norm} (↑)	Lipinski (↑)	Diversity (↑)	Time (s, ↓)
C.D.	1	500	-5.830 ± 2.47	0.403 ± 0.18	0.552 ± 0.13	4.620 ± 0.81	0.808 ± 0.06	97.434 ± 39.79
	5	100	-6.872 ± 2.43	0.444 ± 0.19	0.551 ± 0.12	4.654 ± 0.72	0.766 ± 0.06	96.205 ± 39.22
	10	50	-7.177 ± 3.28	0.556 ± 0.20	0.729 ± 0.12	4.742 ± 0.59	0.718 ± 0.07	94.481 ± 38.86
B.M.	1	500	-5.810 ± 2.00	0.468 ± 0.16	0.627 ± 0.14	4.839 ± 0.49	0.851 ± 0.04	40.298 ± 13.52
	5	100	-6.082 ± 2.01	0.537 ± 0.16	0.701 ± 0.13	4.924 ± 0.31	0.855 ± 0.05	45.074 ± 21.14
	10	50	-6.192 ± 2.24	0.560 ± 0.16	0.737 ± 0.13	4.941 ± 0.27	0.859 ± 0.05	41.490 ± 14.32

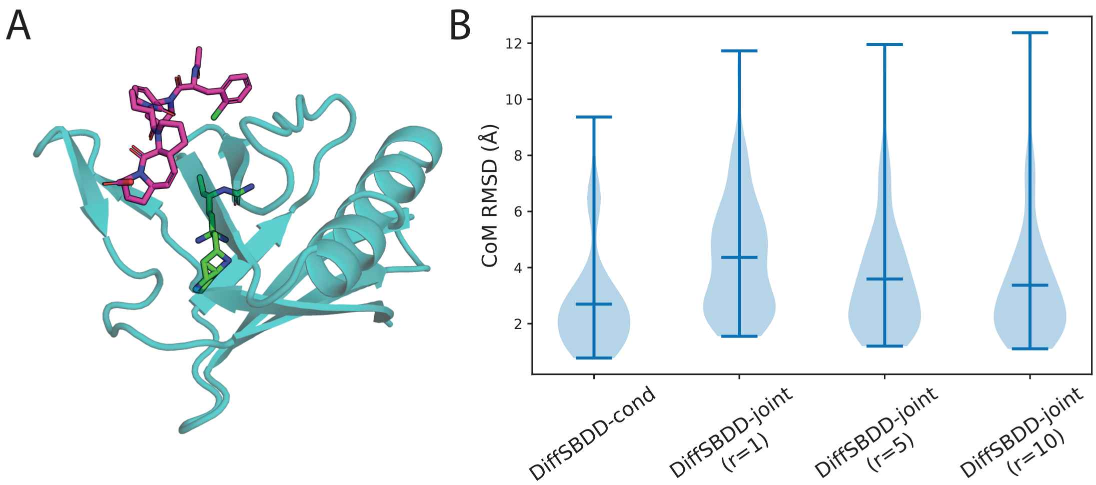

Supplementary Figure 1: Effect of resampling steps for DiffSBDD-joint. (A) Example of a generated molecule (green) without additional resampling steps and the reference molecule (magenta) from the target PDB 5ncf. The generated molecule is not placed in the target pocket but in the protein core. (B) RMSD between reference molecules' center of mass and generated molecules' center of mass for the conditional model and inpaining model with varying numbers of resampling steps r. The pocket representation is C_α in all cases. Minimum, maximum and mean are indicated with horizontal lines. (n=130 in each violin)

Supplementary Section 5.5: Quantitative performance of substructure-constrained models

Supplementary Table 6 reports quantitative results obtained with our molecular inpainting strategy. As baselines, we provide the metrics for the molecules in the test set, molecules only conditioned on the pocket with no fixed substructure (DiffSBDD-*baseline*) and the performance of DiffLinker [19] (the latter for fragment linking). Due to the fact that we can only calculate fragment and scaffold masks for larger molecules, we have reduced the size of the test set used in Supplementary Table 6 to $n = 55$ to ensure fair comparison between methods.

Constraining fixed regions to highly complementary substructures within the protein pocket substantially enhances Vina scores using DiffSBDD-*de novo* compared to the DiffSBDD-*baseline*. For molecules generated without substructure conditioning under DiffSBDD-*baseline*, the average Vina score is -5.69. In contrast, DiffSBDD-*de novo* sees improved scores, achieving -7.74 when used for linker design from starting fragments and achieves results comparable to the specialist model DifLinker. In the case of scaffold elaboration, DiffSBDD-*de novo* substantially boosts docking scores from -5.69 to -8.10 over the baseline, by focusing on the optimal placement of functional groups to facilitate key residue binding on a pre-existing scaffold. Moreover, there is a notable improvement in average docking scores for scaffold hopping, with scores rising from -5.69 to -7.60 when using DiffSBDD-*de novo*. This enhancement is achieved despite a relatively small proportion of atoms being fixed, about 27.32%, compared to other tasks. The substantial increase in docking scores is attributed to the nature of the fixed atoms, primarily functional groups that form the pharmacophore, which are crucial for binding affinity.

Supplementary Table 6: Evaluation of molecular inpainting for fragment linking, scaffold hopping and scaffold elaboration across the Binding MOAD test set. Percentage value next to task name denotes the proportion of atoms fixed during the design process. DiffSBDD-*baseline* generates a whole new molecule from scratch, DiffSBDD-*diversify* is elaborating around a fixed substructure of an existing molecule and DiffSBDD-*de novo* is designing new motifs around a fixed substructure from stratch. t and r are the number of partial noising steps or resamplings for *diversify* and *de novo* respectively.

	Vina (↓)	Validity (↑)	Connectivity (↑)	QED (↑)	SA (↓)	Diversity (↑)
Test set	-9.86 ± 1.6	1	1	0.543 ± 0.16	3.86 ± 1.2	—
DiffSBDD-*baseline*	-5.69 ± 6	0.964	0.589	0.419 ± 0.2	4.99 ± 1.1	0.701 ± 0.094
**Fragment linking (73.43% atoms fixed)**						
DiffSBDD-*de novo* ($r = 20$)	-7.74 ± 1.8	0.796	0.558	0.322 ± 0.17	5.38 ± 0.76	0.486 ± 0.09
DiffSBDD-*diversify* ($t = 100$)	-8.72 ± 1.7	0.991	0.968	0.476 ± 0.14	4.26 ± 1.1	0.35 ± 0.089
DiffLinker [19]	-6.92 ± 2.7	0.947	0.84	0.349 ± 0.19	4.72 ± 0.97	0.453 ± 0.13
**Scaffold hopping (27.32% atoms fixed)**						
DiffSBDD-*de novo* ($r = 20$)	-7.6 ± 2.5	0.782	0.663	0.39 ± 0.18	5.29 ± 0.72	0.612 ± 0.074
DiffSBDD-*diversify* ($t = 100$)	-8.95 ± 1.8	0.977	0.948	0.492 ± 0.18	4.39 ± 1	0.479 ± 0.1
**Scaffold elaboration (72.68% atoms fixed)**						
DiffSBDD-*de novo* ($r = 20$)	-8.1 ± 1.8	0.852	0.445	0.388 ± 0.2	5.2 ± 0.7	0.397 ± 0.11
DiffSBDD-*diversify* ($t = 100$)	-9.32 ± 1.7	0.995	0.971	0.516 ± 0.18	4.12 ± 1.1	0.282 ± 0.14

Supplementary Section 5.6: Importance of resampling for molecular substructure inpainting

Similar to earlier findings, the replacement method often produces poor and inconsistent outcomes. Following the approach by Lugmayr et al. [17], we enhanced the sample quality by refining intermediate states iteratively before progressing in the denoising process, a technique called resampling, as detailed in Methods Section 2.4. This technique is crucial for seamlessly integrating modified and original areas and proves essential in the molecular context. Minimal resampling resulted in chemically valid but disjointed structures, while increased iterations led to coherent molecules, even in complex scenarios with extensive modifications needed. Our results indicate that the effect of resampling on molecular connectivity is particularly pronounced but it also substantially impacts another metrics (Supplementary Figure 2).

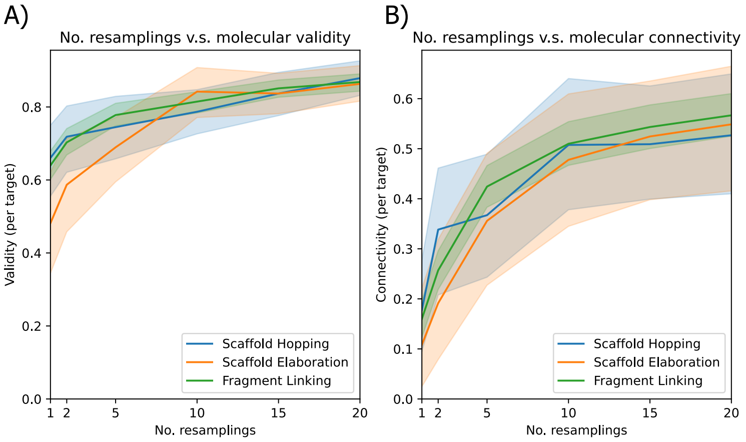

Supplementary Figure 2: Importance of high resamplings. Effect of the number of resamplings on molecular validity (A), connectivity (B) and uniqueness (C). Means and 95% confidence intervals are plotted for 3 design tasks. For this experiment we used 20 randomly selected targets from the test set.

Supplementary Section 5.7: Diversification of candidate molecules

We demonstrate the effect the number of noising/denoising steps (t) has on various molecular properties in Supplementary Figure 3. We test all values of t at intervals of 10 steps and 200 molecules are sampled at every timestep. Note this does not allow for explicit optimization of any particular property unless combined with the evolutionary algorithm, as shown in main text Figure 1D. All plots are for PDB entry 5ndu [20].

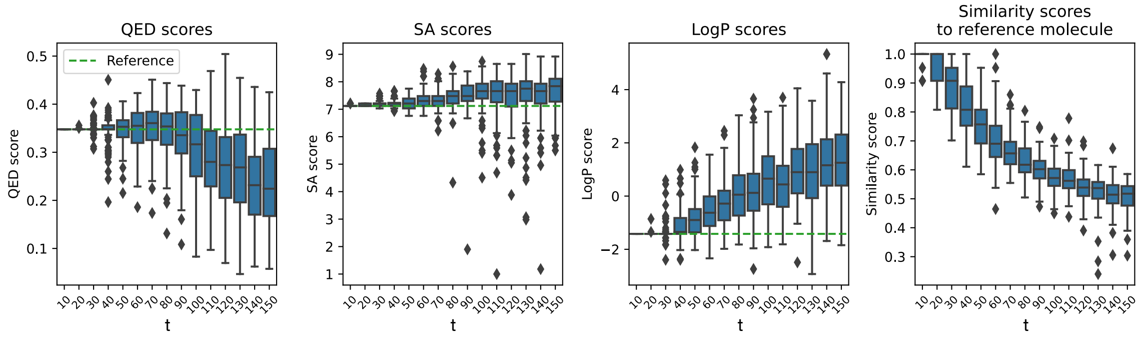

Supplementary Figure 3: Effect of number of noising/denoising steps on molecule properties. Boxes represent the upper and lower quartile as well as the median of the data. Whiskers denote 1.5 times the interquartile range. Outliers outside this range are shown as flier points. Each box represents 100 molecules. *QED*: Quantitative Estimation of Drug-likeness; *SA*: Synthetic Accessibility; *LogP*: is a measure of a molecule's lipophilicity or hydrophobicity; *Sim.*: Tanimoto molecular fingerprint similarity to the reference.

Supplementary Section 5.8: Dependence of Vina scores on molecule size

Supplementary Figure 4 shows how strongly the empirical Vina score is correlated with the number of heavy atoms in the ligands. For this analysis, we used Vina scores computed by the QuickVina2 [21] software after re-docking. Since we want to match the distribution of scores of reference molecules as closely as possible with our generated molecules, we expect that their sizes should roughly match as well. However, our diffusion model operates on point clouds with fixed sizes, which we determine at the beginning of sampling as explained in Methods Section 2.5. By biasing the procedure, we match the sizes of reference ligands more closely as shown in Supplementary Table 7.

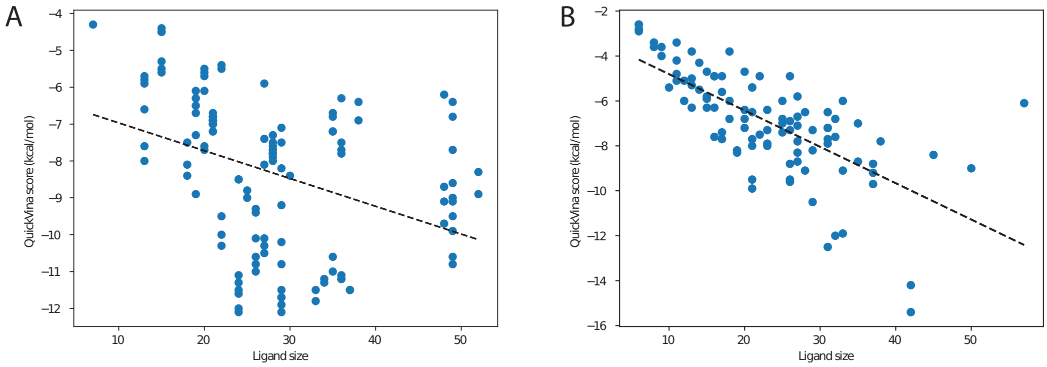

Supplementary Figure 4: Correlation between ligand size and QuickVina score for reference molecules from the Binding MOAD (A) and CrossDocked (B) test sets.

Supplementary Table 7: Average number of heavy atoms of generated molecules.

Method	CrossDocked	Binding MOAD
Test set	23.8	28.0
Pocket2Mol [9]	19.0	16.8
ResGen [10]	16.2	18.0
PocketFlow [15]	19.8	19.3
DeepICL [16]	20.9	–
DiffSBDD-cond	24.8	24.4
DiffSBDD-joint	24.5	25.2

Supplementary Section 5.9: Results with a coarse-grained pocket representation

Here we discuss the advantages and disadvantages of coarse-grained C_α protein representations as context for the generative model. Supplementary Table 8 shows that full-atom models outperform their coarse-grained counterparts on the Vina metric, which is the only reported metric that captures interactions with the protein. Ligand-centric metrics do not seem to depend on the protein representation as could be expected. The main advantage of the C_α models is their substantially faster training and inference time, a fact we made use of during model development for fine-tuning and preliminary analyses.

To further demonstrate the limitations of the coarse-grained models, we compare the generated raw conformations to the best scoring QuickVina docking pose after re-docking and plot the distribution of resulting RMSD values in Supplementary Figure 5. As a baseline, the procedure is repeated for RDKit conformers of the same molecules with identical center of mass. For a large percentage of molecules generated by the all-atom models, QuickVina agrees with the predicted bound conformations, leaving them almost unchanged (RMSD below 2 Å). This demonstrates successful conditioning on the geometry of the given protein pockets. For the C_α-only models results are less convincing. They produce poses that barely improve upon conformers lacking pocket-context. Likely, this is caused by atomic clashes with the proteins' side chains that QuickVina needs to resolve.

Supplementary Table 8: Evaluation of generated molecules for targets from the CrossDocked (C.D.) and Binding MOAD (B.M.) test sets. We compare all-atom and coarse-grained (C_α) pocket representations. Here, the SA scores were mapped to the unit interval using $\text{SA}_{\text{norm}} = (10 - \text{SA})/9$.

		Vina (All) (↓)	Vina (Top-10%) (↓)	QED (↑)	SA_{norm} (↑)	Lipinski (↑)	Diversity (↑)	Time (s, ↓)
C.D.	Test set	-6.871 ± 2.32	—	0.476 ± 0.20	0.728 ± 0.14	4.340 ± 1.14	—	—
	DiffSBDD-cond (C_α)	-6.770 ± 2.73	-8.796 ± 1.75	0.475 ± 0.22	0.612 ± 0.12	4.536 ± 0.91	0.725 ± 0.06	49.651 ± 17.34
	DiffSBDD-joint (C_α)	-7.177 ± 3.28	-9.233 ± 1.82	0.556 ± 0.20	0.729 ± 0.12	4.742 ± 0.59	0.718 ± 0.07	94.481 ± 38.86
	DiffSBDD-cond	-6.950 ± 2.06	-9.120 ± 2.16	0.469 ± 0.21	0.578 ± 0.13	4.562 ± 0.89	0.728 ± 0.07	135.866 ± 51.66
	DiffSBDD-joint	-7.333 ± 2.56	-9.927 ± 2.59	0.467 ± 0.18	0.554 ± 0.12	4.702 ± 0.64	0.758 ± 0.05	160.314 ± 73.30
B.M.	Test set	-8.412 ± 2.03	—	0.522 ± 0.17	0.692 ± 0.12	4.669 ± 0.49	—	—
	DiffSBDD-cond (C_α)	-6.863 ± 1.59	-8.587 ± 1.34	0.480 ± 0.20	0.554 ± 0.11	4.662 ± 0.68	0.714 ± 0.05	36.285 ± 8.13
	DiffSBDD-joint (C_α)	-6.926 ± 3.39	-9.124 ± 1.35	0.548 ± 0.19	0.580 ± 0.13	4.757 ± 0.51	0.709 ± 0.05	58.305 ± 17.35
	DiffSBDD-cond	-7.171 ± 1.89	-9.184 ± 2.23	0.436 ± 0.20	0.568 ± 0.12	4.542 ± 0.79	0.714 ± 0.08	336.061 ± 85.02
	DiffSBDD-joint	-7.309 ± 4.03	-9.840 ± 2.18	0.542 ± 0.21	0.615 ± 0.12	4.777 ± 0.53	0.739 ± 0.05	369.873 ± 124.54

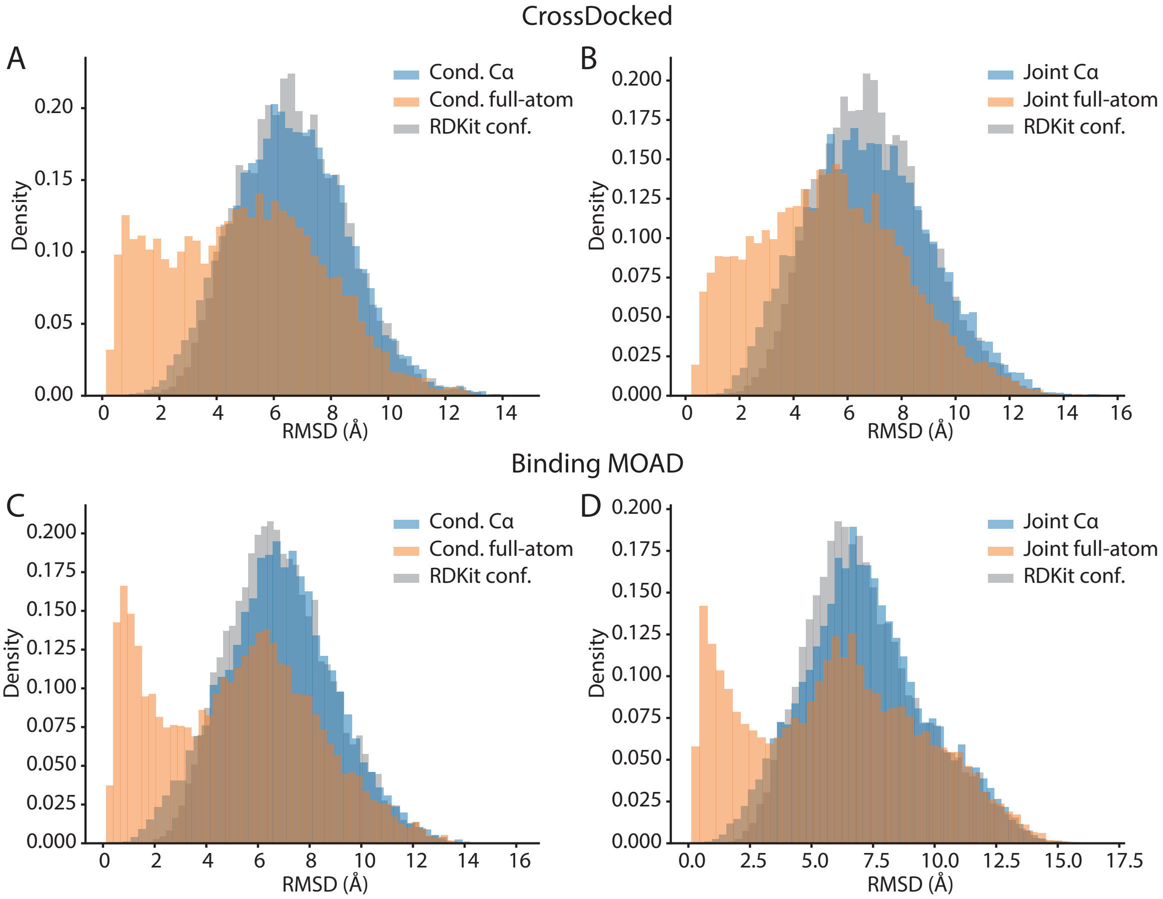

Supplementary Figure 5: RMSD between generated and docked conformations for the CrossDocked (A, B) and Binding MOAD (C, D) datasets. Full-atom models are compared to C_α models as well as a baseline of random RDKit conformers of the molecules generated by the C_α-model. (A, C) DiffSBDD-cond. (B, D) DiffSBDD-joint.

Supplementary Section 6: Supplementary Tables

Supplementary Section 6.1: Sampling statistics for the distribution learning benchmark

Supplementary Table 9: Average number of molecules per test set pocket in the *de novo* design experiment.

Method	CrossDocked	Binding MOAD
Test set	1.0	1.0
Pocket2Mol [9]	98.0	132.1
ResGen [10]	114.5	109.9
PocketFlow [15]	100.0	100.0
DeepICL [16]	100.0	–
DiffSBDD-cond	100.0	100.0
DiffSBDD-joint	100.0	100.0

Supplementary Section 6.2: Sampling statistics for the substructure design experiment

Supplementary Table 10: Sampling statistics for the substructure design experiment. 'Num. pockets' is the number of pockets for which we could sample and evaluate at least one molecule.

		Molecules per pocket			
	Num. pockets	Mean	Std. Dev.	Min	Max
Test set	55	1.0	0.0	1	1
DiffSBDD-*baseline*	55	125.3	2.2	120	130
**Fragment linking**					
DiffSBDD-*de novo*	55	915.1	527.6	32	2150
DiffSBDD-*diversify*	55	1138.4	641.6	100	2497
DiffLinker [19]	55	428.9	393.1	87	899
**Scaffold hopping**					
DiffSBDD-*de novo*	55	78.2	23.3	21	100
DiffSBDD-*diversify*	55	97.7	2.9	89	100
**Scaffold elaboration**					
DiffSBDD-*de novo*	55	85.2	17.3	20	100
DiffSBDD-*diversify*	55	99.5	1.0	95	100

Supplementary Section 6.3: Training hyperparameters

Hyperparameters for all presented models are summarized in Supplementary Table 11. Training takes about 2.5 h/3.8 h (conditional/joint) per 100 epochs on a single NVIDIA V100 for Binding MOAD in the C_α scenario and 11.5 h/14.7 h per 100 epochs with full atom pocket representation on two V100 GPUs. For CrossDocked, 100 training epochs take approximately 6 h/8 h in the C_α case and 48 h/60 h per 100 epochs on a single NVIDIA A100 GPU with all atom pocket representation.

Supplementary Table 11: DiffSBDD hyperparameters.

	CrossDocked				Binding MOAD			
	Cond	Joint	Cond (C_α)	Joint (C_α)	Cond	Joint	Cond (C_α)	Joint (C_α)
No. layers	5	5	6	6	6	6	5	5
Joint embedding dim.	32	32	128	128	128	128	32	32
Hidden dim.	128	128	256	256	192	192	128	128
Learning rate	10^{-3}	10^{-3}	10^{-3}	10^{-3}	$5 \cdot 10^{-4}$	$5 \cdot 10^{-4}$	$5 \cdot 10^{-4}$	$5 \cdot 10^{-4}$
Weight decay	10^{-12}	10^{-12}	10^{-12}	10^{-12}	10^{-12}	10^{-12}	10^{-12}	10^{-12}
Diffusion steps	500	500	500	500	500	500	500	500
Edges (ligand-ligand)	fully connected	fully connected	fully connected	fully connected	fully connected	fully connected	fully connected	fully connected
Edges (ligand-pocket)	< 5 Å	< 5 Å	< 5 Å	< 5 Å	< 7 Å	< 7 Å	< 8 Å	< 8 Å
Edges (pocket-pocket)	< 5 Å	< 5 Å	< 5 Å	< 5 Å	< 4 Å	< 4 Å	< 8 Å	< 8 Å
Epochs	1000	1000	1000	1000	1000	1000	1000	1000

Supplementary Section 6.4: Replacement method algorithm

Algorithm 1 Sampling with the replacement method and resampling iterations. r denotes the number of resampling steps and $\mathcal{M}$ is a set of indices of all atoms we want to fix. Note that samples from the generative process $p_\theta(\boldsymbol{z}_{t-1}|\boldsymbol{z}_t)$ are assumed to be CoM-free.

Require: r, $\mathcal{M}$
  $\boldsymbol{z}_T \sim \mathcal{N}(\mathbf{0}, \boldsymbol{I})$
  for $t = T, ..., 1$ **do**
    for $k = 1, ..., r$ **do**
      $\boldsymbol{z}_{t-1}^{\text{input}} \sim q(\boldsymbol{z}_{t-1}|\boldsymbol{z}_{\text{data}})$ ▷ Sample known context
      $\boldsymbol{z}_{t-1}^{\text{gen}} \sim p_\theta(\boldsymbol{z}_{t-1}|\boldsymbol{z}_t)$ ▷ Sample generated part
      $\tilde{\boldsymbol{x}}_{t-1}^{\text{input}} = \boldsymbol{x}_{t-1}^{\text{input}} + \frac{1}{n}\sum_{i\in\mathcal{M}} \boldsymbol{x}_{t-1,i}^{\text{gen}} - \frac{1}{n}\sum_{i\in\mathcal{M}} \boldsymbol{x}_{t-1,i}^{\text{input}}$ ▷ Adjust center of mass
      $\boldsymbol{z}_{t-1} = [\tilde{\boldsymbol{z}}_{t-1}^{\text{input}}, \boldsymbol{z}_{t-1,i\notin\mathcal{M}}^{\text{gen}}]$ ▷ Combine
      if $k < r$ **then**
        $\boldsymbol{z}_t \sim q(\boldsymbol{z}_t|\boldsymbol{z}_{t-1})$ ▷ Apply noise and repeat
      end if
    end for
  end for
  return $\boldsymbol{z}_0$

Supplementary Section 7: Supplementary Figures

Supplementary Section 7.1: Distributions of molecular properties

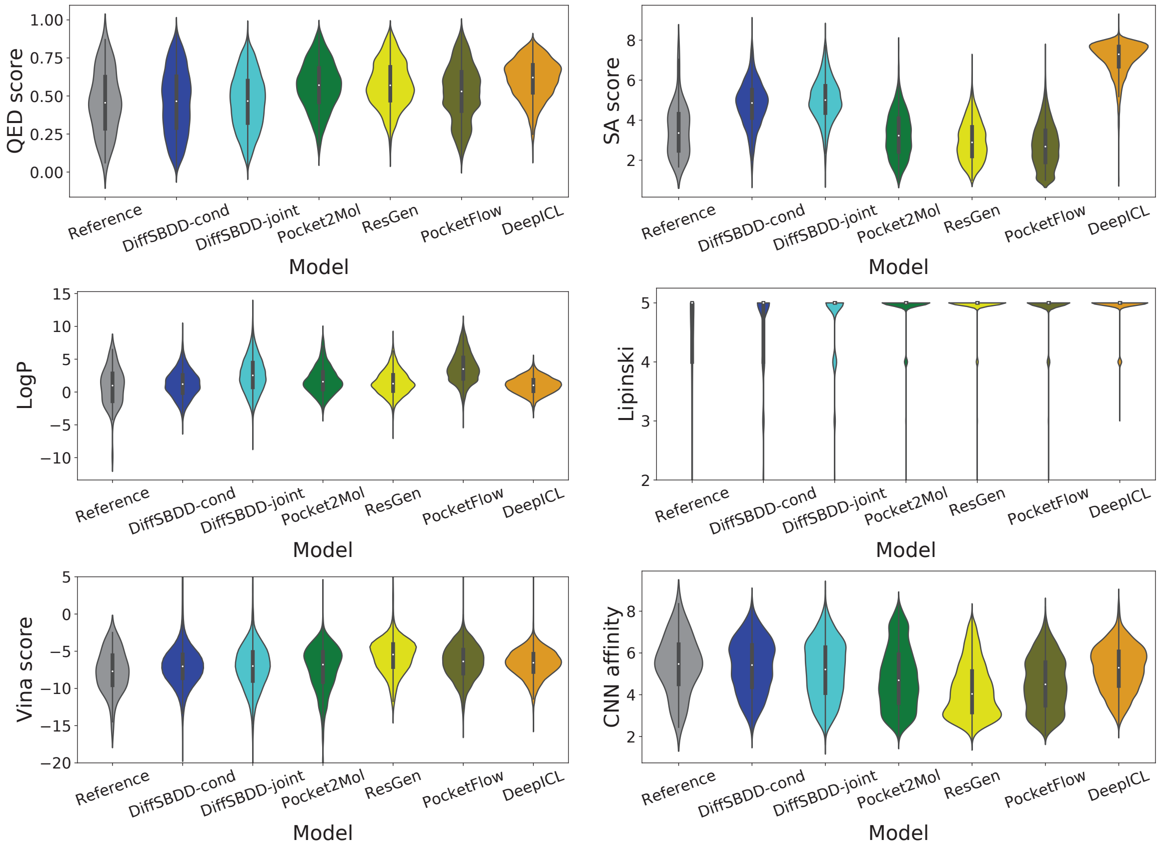

Supplementary Figure 6: Distributions of computational scores for generated molecules and reference ligands from the CrossDocked test set. All box plots within violins include the median line, a box denoting the interquartile range (IQR) and whiskers showing data within $\pm 1.5 \times$ IQR. (n=78, 7800, 7800, 7643, 8932, 7800, 7800 for Reference, DiffSBDD-cond, DiffSBDD-joint, Pocket2Mol, ResGen, PocketFlow, DeepICL, respectively)

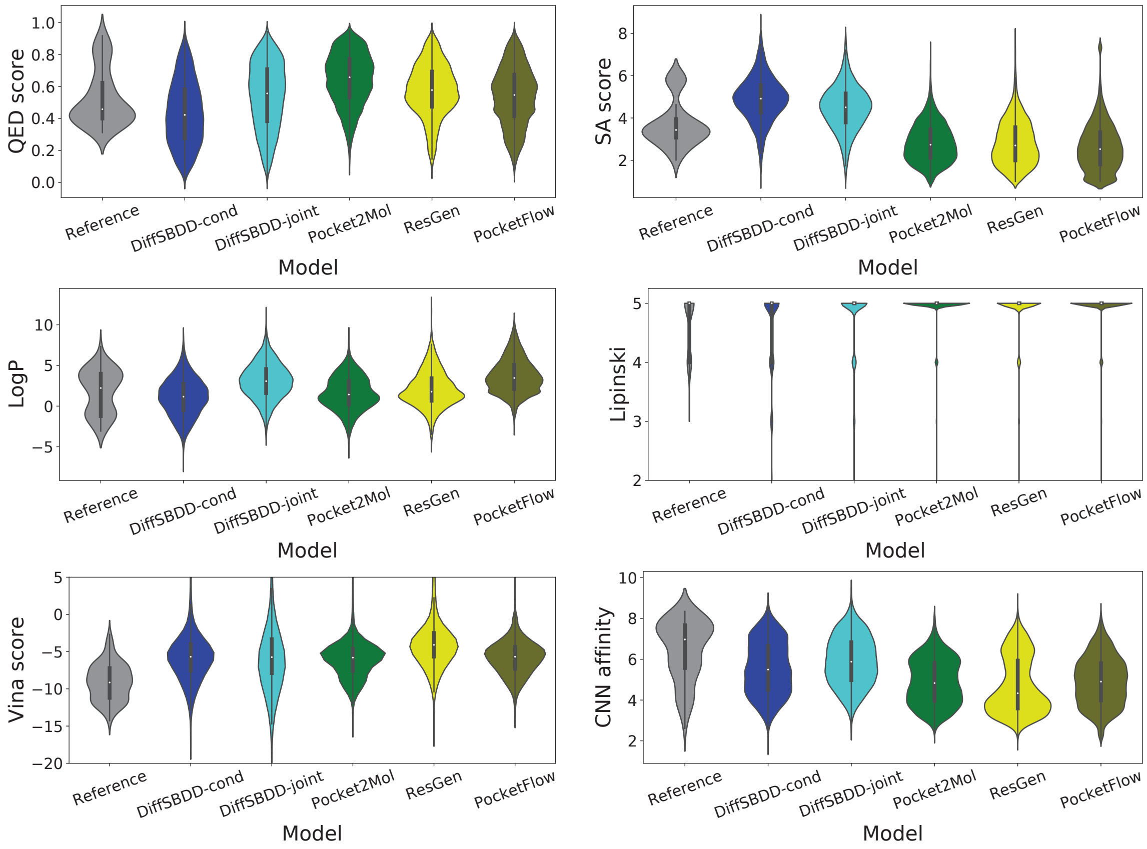

Supplementary Figure 7: Distributions of computational scores for generated molecules and reference ligands from the Binding MOAD test set. All box plots within violins include the median line, a box denoting the interquartile range (IQR) and whiskers showing data within $\pm 1.5 \times$ IQR. (n=119, 11 900, 11 900, 15 718, 13 074, 11 900 for Reference, DiffSBDD-cond, DiffSBDD-joint, Pocket2Mol, ResGen, PocketFlow, respectively)

Supplementary Section 7.2: Generated molecules

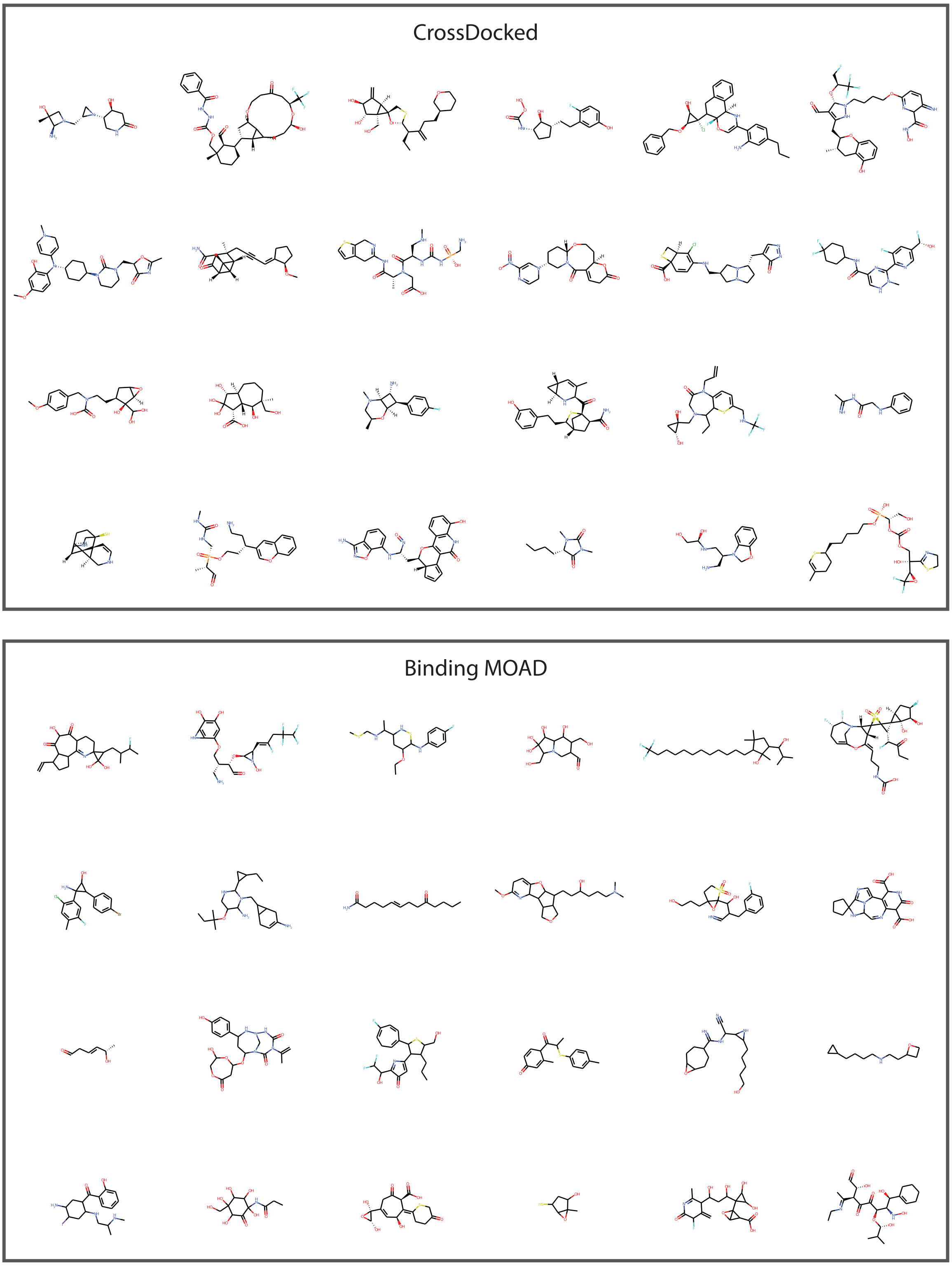

Supplementary Figure 8: Randomly selected samples of molecules generated by DiffSBDD-cond.

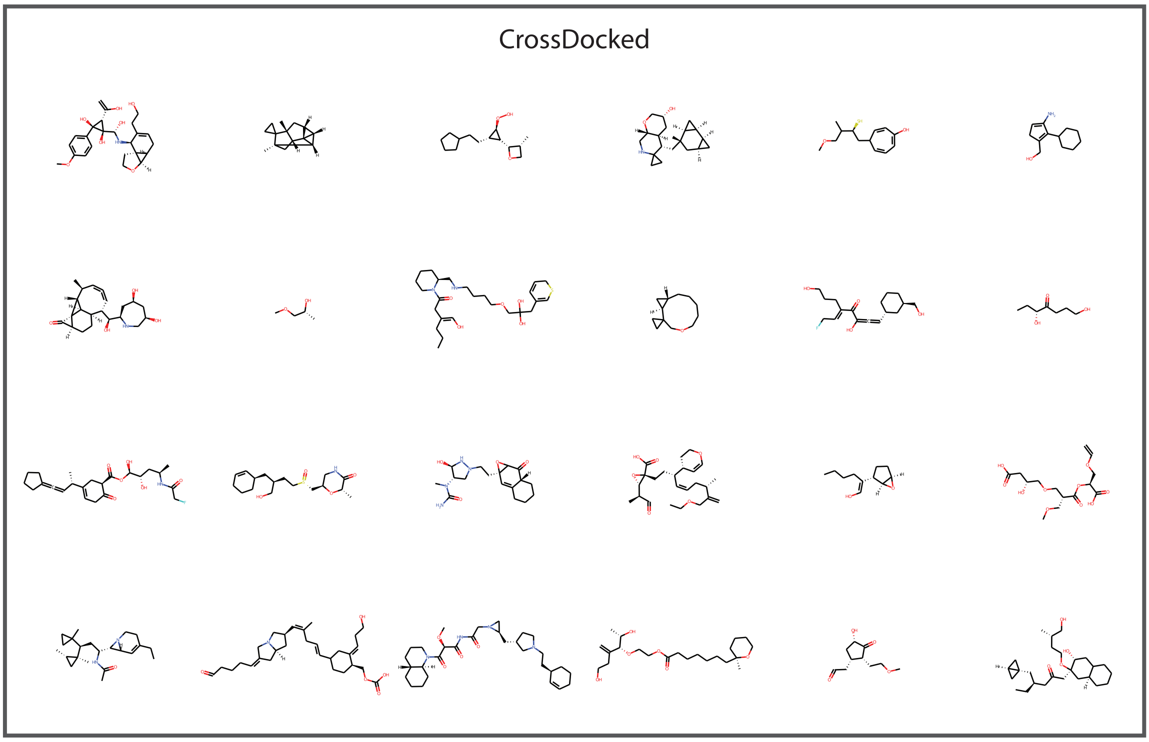

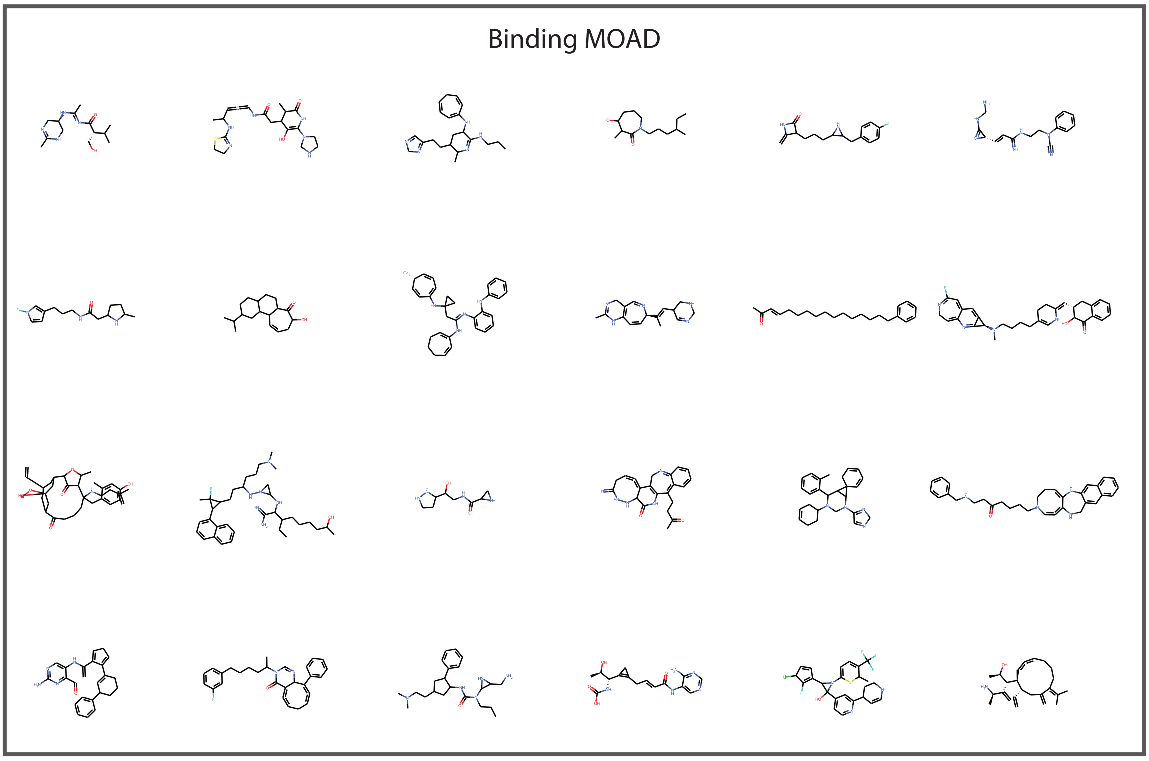

Supplementary Figure 9: Randomly selected samples of molecules generated by DiffSBDD-joint.

Supplementary Section 8: Related Work

Diffusion Models for Molecules

Inspired by non-equilibrium thermodynamics, diffusion models have been proposed to learn data distributions by modeling a denoising (reverse diffusion) process and have achieved remarkable success in a variety of tasks such as image, audio synthesis and point cloud generation [1, 22, 23]. Recently, efforts have been made to utilize diffusion models for molecule design [24]. Specifically, Hoogeboom et al. [2] propose a diffusion model with an equivariant network that operates both on continuous atomic coordinates and categorical atom types to generate new molecules in 3D space. Torsional Diffusion [25] focuses on a conditional setting where molecular conformations (atomic coordinates) are generated from molecular graphs (atom types and bonds). Similarly, 3D diffusion models have been applied to generative design of larger biomolecular structures, such as antibodies [26] and other proteins [27, 28].

Structure-based Drug Design

Structure-based Drug Design (SBDD) [29, 30] relies on the knowledge of the 3D structure of the biological target obtained either through experimental methods or high-confidence predictions using homology modelling [31]. Candidate molecules are then designed to bind with high affinity and specificity to the target using interactive software [32] and often human-based intuition [29]. Recent advances in deep generative models have brought a new wave of research that model the conditional distribution of ligands given biological targets and thus enable *de novo* structure-based drug design. Most of previous work consider this task as a sequential generation problem and design a variety of generative methods including autoregressive models, reinforcement learning, etc., to generate ligands inside protein pockets atom by atom [9, 33–35]. Most recent work explore the use of diffusion models in structure-based drug design [36–38].

Geometric Deep Learning for Drug Discovery

Geometric deep learning refers to incorporating geometric priors in neural architecture design that respects symmetry and invariance, thus reduces sample complexity and eliminates the need for data augmentation [39]. It has been prevailing in a variety of drug discovery tasks from virtual screening to *de novo* drug design as symmetry widely exists in the representation of drugs. One line of work introduces graph and geometry priors and designs message passing neural networks and equivariant neural networks that are permutation-, translation-, rotation-, and reflection-equivariant, respectively [3, 40–43]. These architectures have been widely used in representing biomolecules from small molecules to proteins [44] and solving downstream tasks such as molecular property prediction [45, 46], binding pose prediction [47], transition state sampling [48], and molecular dynamics [49, 50]. Another line of work focuses on generative design of new molecules [24]. More specifically, molecule design is formulated as a graph or geometry generation problem, following either a one-shot generation strategy that generates graphs (atom and bond features) in one step or attempting to generate atoms and bonds sequentially.

References

[1] Kingma, D., Salimans, T., Poole, B., Ho, J.: Variational diffusion models. Advances in neural information processing systems **34**, 21696–21707 (2021)

[2] Hoogeboom, E., Satorras, V.G., Vignac, C., Welling, M.: Equivariant diffusion for molecule generation in 3d. In: International Conference on Machine Learning, pp. 8867–8887 (2022). PMLR

[3] Satorras, V.G., Hoogeboom, E., Welling, M.: E (n) equivariant graph neural networks. In: International Conference on Machine Learning, pp. 9323–9332 (2021). PMLR

[4] Adams, K., Pattanaik, L., Coley, C.W.: Learning 3d representations of molecular chirality with invariance to bond rotations. arXiv preprint arXiv:2110.04383 (2021)

[5] McNutt, A.T., Francoeur, P., Aggarwal, R., Masuda, T., Meli, R., Ragoza, M., Sunseri, J., Koes, D.R.: Gnina 1.0: molecular docking with deep learning. Journal of cheminformatics **13**(1), 1–20 (2021)

[6] Francoeur, P.G., Masuda, T., Sunseri, J., Jia, A., Iovanisci, R.B., Snyder, I., Koes, D.R.: Three-dimensional convolutional neural networks and a cross-docked data set for structure-based drug design. Journal of Chemical Information and Modeling **60**(9), 4200–4215 (2020)

[7] Ragoza, M., Masuda, T., Koes, D.R.: Generating 3d molecules conditional on receptor binding sites with deep generative models. Chemical science **13**(9), 2701–2713 (2022)

[8] Liu, M., Luo, Y., Uchino, K., Maruhashi, K., Ji, S.: Generating 3d molecules for target protein binding. arXiv preprint arXiv:2204.09410 (2022)

[9] Peng, X., Luo, S., Guan, J., Xie, Q., Peng, J., Ma, J.: Pocket2mol: Efficient molecular sampling based on 3d protein pockets. arXiv preprint arXiv:2205.07249 (2022)

[10] Zhang, O., Zhang, J., Jin, J., Zhang, X., Hu, R., Shen, C., Cao, H., Du, H., Kang, Y., Deng, Y., et al.: Resgen is a pocket-aware 3d molecular generation model based on parallel multiscale modelling. Nature Machine Intelligence, 1–11 (2023)

[11] Hu, L., Benson, M.L., Smith, R.D., Lerner, M.G., Carlson, H.A.: Binding moad (mother of all databases). Proteins: Structure, Function, and Bioinformatics **60**(3), 333–340 (2005)

[12] Bickerton, G.R., Paolini, G.V., Besnard, J., Muresan, S., Hopkins, A.L.: Quantifying the chemical beauty of drugs. Nature chemistry **4**(2), 90–98 (2012)

[13] Ertl, P., Schuffenhauer, A.: Estimation of synthetic accessibility score of drug-like molecules based on molecular complexity and fragment contributions. Journal of cheminformatics **1**(1), 1–11 (2009)

[14] Wildman, S.A., Crippen, G.M.: Prediction of physicochemical parameters by atomic contributions. Journal of chemical information and computer sciences **39**(5), 868–873 (1999)

[15] Jiang, Y., Zhang, G., You, J., Zhang, H., Yao, R., Xie, H., Zhang, L., Xia, Z., Dai, M., Wu, Y., *et al.*: Pocketflow is a data-and-knowledge-driven structure-based molecular generative model. Nature Machine Intelligence **6**(3), 326–337 (2024)

[16] Zhung, W., Kim, H., Kim, W.Y.: 3d molecular generative framework for interaction-guided drug design. Nature Communications **15**(1), 2688 (2024)

[17] Lugmayr, A., Danelljan, M., Romero, A., Yu, F., Timofte, R., Van Gool, L.: Repaint: Inpainting using denoising diffusion probabilistic models. In: Proceedings of the IEEE/CVF Conference on Computer Vision and Pattern Recognition, pp. 11461–11471 (2022)

[18] Nichol, A.Q., Dhariwal, P.: Improved denoising diffusion probabilistic models. In: International Conference on Machine Learning, pp. 8162–8171 (2021). PMLR

[19] Igashov, I., Stärk, H., Vignac, C., Schneuing, A., Satorras, V.G., Frossard, P., Welling, M., Bronstein, M., Correia, B.: Equivariant 3d-conditional diffusion model for molecular linker design. Nature Machine Intelligence, 1–11 (2024)

[20] Barone, M., Müller, M., Chiha, S., Ren, J., Albat, D., Soicke, A., Dohmen, S., Klein, M., Bruns, J., Dinther, M., *et al.*: Designed nanomolar small-molecule inhibitors of ena/vasp evh1 interaction impair invasion and extravasation of breast cancer cells. Proceedings of the National Academy of Sciences **117**(47), 29684–29690 (2020)

[21] Alhossary, A., Handoko, S.D., Mu, Y., Kwoh, C.-K.: Fast, accurate, and reliable molecular docking with quickvina 2. Bioinformatics **31**(13), 2214–2216 (2015)

[22] Kong, Z., Ping, W., Huang, J., Zhao, K., Catanzaro, B.: Diffwave: A versatile diffusion model for audio synthesis. In: International Conference on Learning Representations (2021)

[23] Luo, S., Hu, W.: Diffusion probabilistic models for 3d point cloud generation. In: Proceedings of the IEEE/CVF Conference on Computer Vision and Pattern Recognition, pp. 2837–2845 (2021)

[24] Du, Y., Fu, T., Sun, J., Liu, S.: Molgensurvey: A systematic survey in machine learning models for molecule design. arXiv preprint arXiv:2203.14500 (2022)

[25] Jing, B., Corso, G., Chang, J., Barzilay, R., Jaakkola, T.: Torsional diffusion for molecular conformer generation. arXiv preprint arXiv:2206.01729 (2022)

[26] Luo, S., Su, Y., Peng, X., Wang, S., Peng, J., Ma, J.: Antigen-specific antibody design and optimization with diffusion-based generative models. bioRxiv (2022)

[27] Anand, N., Achim, T.: Protein structure and sequence generation with equivariant denoising diffusion probabilistic models. arXiv preprint arXiv:2205.15019 (2022)

[28] Trippe, B.L., Yim, J., Tischer, D., Broderick, T., Baker, D., Barzilay, R., Jaakkola, T.: Diffusion probabilistic modeling of protein backbones in 3d for the motif-scaffolding problem. arXiv preprint arXiv:2206.04119 (2022)

[29] Ferreira, L.G., Dos Santos, R.N., Oliva, G., Andricopulo, A.D.: Molecular docking and structure-based drug design strategies. Molecules **20**(7), 13384–13421 (2015)

[30] Anderson, A.C.: The process of structure-based drug design. Chemistry & biology **10**(9), 787–797 (2003)

[31] Kelley, L.A., Mezulis, S., Yates, C.M., Wass, M.N., Sternberg, M.J.: The phyre2 web portal for protein modeling, prediction and analysis. Nature protocols **10**(6), 845–858 (2015)

[32] Kalyaanamoorthy, S., Chen, Y.-P.P.: Structure-based drug design to augment hit discovery. Drug discovery today **16**(17-18), 831–839 (2011)

[33] Drotár, P., Jamasb, A.R., Day, B., Cangea, C., Liò, P.: Structure-aware generation of drug-like molecules. arXiv preprint arXiv:2111.04107 (2021)

[34] Luo, S., Guan, J., Ma, J., Peng, J.: A 3d generative model for structure-based drug design. Advances in Neural Information Processing Systems **34**, 6229–6239 (2021)

[35] Li, Y., Pei, J., Lai, L.: Structure-based de novo drug design using 3d deep generative models. Chemical science **12**(41), 13664–13675 (2021)

[36] Guan, J., Qian, W.W., Peng, X., Su, Y., Peng, J., Ma, J.: 3d equivariant diffusion for target-aware molecule generation and affinity prediction. arXiv preprint arXiv:2303.03543 (2023)

[37] Lin, H., Huang, Y., Liu, M., Li, X., Ji, S., Li, S.Z.: Diffbp: Generative diffusion of 3d molecules for target protein binding. arXiv preprint arXiv:2211.11214 (2022)

[38] Guan, J., Zhou, X., Yang, Y., Bao, Y., Peng, J., Ma, J., Liu, Q., Wang, L., Gu, Q.: Decompdiff: Diffusion models with decomposed priors for structure-based drug design (2023)

[39] Bronstein, M.M., Bruna, J., Cohen, T., Veličković, P.: Geometric deep learning: Grids, groups, graphs, geodesics, and gauges. arXiv preprint arXiv:2104.13478 (2021)

[40] Duvenaud, D.K., Maclaurin, D., Iparraguirre, J., Bombarell, R., Hirzel, T., Aspuru-Guzik, A., Adams, R.P.: Convolutional networks on graphs for learning molecular fingerprints. Advances in neural information processing systems **28** (2015)

[41] Gilmer, J., Schoenholz, S.S., Riley, P.F., Vinyals, O., Dahl, G.E.: Neural message passing for quantum chemistry. In: International Conference on Machine Learning, pp. 1263–1272 (2017). PMLR

[42] Lapchevskyi, K., Miller, B., Geiger, M., Smidt, T.: Euclidean neural networks (e3nn) v1. 0. Technical report, Lawrence Berkeley National Lab.(LBNL), Berkeley, CA (United States) (2020)

[43] Du, W., Zhang, H., Du, Y., Meng, Q., Chen, W., Zheng, N., Shao, B., Liu, T.-Y.: Se (3) equivariant graph neural networks with complete local frames. In: International Conference on Machine Learning, pp. 5583–5608 (2022). PMLR

[44] Atz, K., Grisoni, F., Schneider, G.: Geometric deep learning on molecular representations. Nature Machine Intelligence **3**(12), 1023–1032 (2021)

[45] Schütt, K.T., Sauceda, H.E., Kindermans, P.-J., Tkatchenko, A., Müller, K.-R.: Schnet–a deep learning architecture for molecules and materials. The Journal of Chemical Physics **148**(24), 241722 (2018)

[46] Klicpera, J., Groß, J., Günnemann, S.: Directional message passing for molecular graphs. arXiv preprint arXiv:2003.03123 (2020)

[47] Stärk, H., Ganea, O., Pattanaik, L., Barzilay, R., Jaakkola, T.: Equibind: Geometric deep learning for drug binding structure prediction. In: International Conference on Machine Learning, pp. 20503–20521 (2022). PMLR

[48] Duan, C., Du, Y., Jia, H., Kulik, H.J.: Accurate transition state generation with an object-aware equivariant elementary reaction diffusion model. Nature Computational Science **3**(12), 1045–1055 (2023)

[49] Batzner, S., Musaelian, A., Sun, L., Geiger, M., Mailoa, J.P., Kornbluth, M., Molinari, N., Smidt, T.E., Kozinsky, B.: E (3)-equivariant graph neural networks for data-efficient and accurate interatomic potentials. Nature communications **13**(1), 1–11 (2022)

[50] Holdijk, L., Du, Y., Hooft, F., Jaini, P., Ensing, B., Welling, M.: Path integral stochastic optimal control for sampling transition paths. arXiv preprint arXiv:2207.02149 (2022)
