# Supplementary material for: Structure-based drug design with equivariant diffusion models
Source: Nat Comput Sci. 2024 Dec 9;4(12):899–909. doi: 10.1038/s43588-024-00737-x (PMC11659159; doi:10.1038/s43588-024-00737-x)
Supplement: Supplementary file 2 — Reporting Summary [file 43588_2024_737_MOESM2_ESM.pdf]

nature portfolio

Corresponding author(s): Bruno Correia

Last updated by author(s): Oct 16, 2024

Reporting Summary

Nature Portfolio wishes to improve the reproducibility of the work that we publish. This form provides structure for consistency and transparency in reporting. For further information on Nature Portfolio policies, see our Editorial Policies and the Editorial Policy Checklist.

Statistics

For all statistical analyses, confirm that the following items are present in the figure legend, table legend, main text, or Methods section.

n/a	Confirmed	
☐	☒	The exact sample size (*n*) for each experimental group/condition, given as a discrete number and unit of measurement
☒	☐	A statement on whether measurements were taken from distinct samples or whether the same sample was measured repeatedly
☒	☐	The statistical test(s) used AND whether they are one- or two-sided<br>*Only common tests should be described solely by name; describe more complex techniques in the Methods section.*
☒	☐	A description of all covariates tested
☒	☐	A description of any assumptions or corrections, such as tests of normality and adjustment for multiple comparisons
☐	☒	A full description of the statistical parameters including central tendency (e.g. means) or other basic estimates (e.g. regression coefficient) AND variation (e.g. standard deviation) or associated estimates of uncertainty (e.g. confidence intervals)
☒	☐	For null hypothesis testing, the test statistic (e.g. *F*, *t*, *r*) with confidence intervals, effect sizes, degrees of freedom and *P* value noted<br>*Give P values as exact values whenever suitable.*
☒	☐	For Bayesian analysis, information on the choice of priors and Markov chain Monte Carlo settings
☒	☐	For hierarchical and complex designs, identification of the appropriate level for tests and full reporting of outcomes
☒	☐	Estimates of effect sizes (e.g. Cohen's *d*, Pearson's *r*), indicating how they were calculated

Our web collection on statistics for biologists contains articles on many of the points above.

Software and code

Policy information about availability of computer code

Data collection	All code was written in Python (v3.10.4). For dataset preparation, we used numpy (v1.22.4), BioPython (v1.81) and RDKit (v2023.9.4). The neural network models were implemented and trained with PyTorch (v1.12.1), PyTorch Lightning (v1.7.4), PyTorch Geometric (v2.2) and Weights & Biases (v0.13.1). OpenBabel (v3.1.1) and RDKit (v2023.9.4) were used to post-process molecules. Docking/scoring was performed using the Gnina (v1.1) and QuickVina (v2.1) softwares.<br>Our source codes are available at https://github.com/arneschneuing/DiffSBDD. Model weights can be downloaded from Zenodo (https://zenodo.org/records/8183747).<br><br>The code required to run the baseline models is also available in public repositories. Pocket2Mol can be found at https://github.com/pengxingang/Pocket2Mol, ResGen at https://github.com/HaotianZhangAI4Science/ResGen, PocketFlow (latest) at https://github.com/Saoge123/PocketFlow, and DeepICL (v1.1.0) at https://github.com/ACE-KAIST/DeepICL. Finally, DiffLinker (v1.0) is available at https://github.com/igashov/DiffLinker. The Pocket2Mol and ResGen repositories do not provide version releases.
Data analysis	In addition to the packages mentioned above, the data was analyzed using Pandas (v1.4.2), SciPy (v1.7.3), Matplotlib (v3.4.3) and Seaborn (v0.12.0).

For manuscripts utilizing custom algorithms or software that are central to the research but not yet described in published literature, software must be made available to editors and reviewers. We strongly encourage code deposition in a community repository (e.g. GitHub). See the Nature Portfolio guidelines for submitting code & software for further information.

Data

Policy information about availability of data

All manuscripts must include a data availability statement. This statement should provide the following information, where applicable:

- Accession codes, unique identifiers, or web links for publicly available datasets
- A description of any restrictions on data availability
- For clinical datasets or third party data, please ensure that the statement adheres to our policy

We use a subset of the CrossDocked dataset (https://github.com/gnina/models/tree/master/data/CrossDocked2020) that was curated in a previous work and is available online: https://github.com/pengxingang/Pocket2Mol/tree/main/data. The raw BindingMOAD data can be downloaded from http://www.bindingmoad.org/. Processed versions of these datasets are available on Zenodo (https://doi.org/10.5281/zenodo.13931612).
We also provide sampled molecules on Zenodo (https://doi.org/10.5281/zenodo.8239058).

Structural models of the protein targets discussed in this study are available under PDB accession codes: 2buj, 2gm1, 4tos, 4w9w, 5ndu, 5rsw, 5rue, 5spd, 6c0b, 6rcj. The starting molecule from the selective kinase design experiment has ChEMBL identifier CHEMBL388978.

Human research participants

Policy information about studies involving human research participants and Sex and Gender in Research.

Reporting on sex and gender	n/a
Population characteristics	n/a
Recruitment	n/a
Ethics oversight	n/a

Note that full information on the approval of the study protocol must also be provided in the manuscript.

Field-specific reporting

Please select the one below that is the best fit for your research. If you are not sure, read the appropriate sections before making your selection.

☒ Life sciences ☐ Behavioural & social sciences ☐ Ecological, evolutionary & environmental sciences

For a reference copy of the document with all sections, see nature.com/documents/nr-reporting-summary-flat.pdf

Life sciences study design

All studies must disclose on these points even when the disclosure is negative.

Sample size	The number of target proteins in our test sets follows previous studies that used 100 protein pockets from the CrossDocked dataset (Peng et. al., 2022). We aimed to sample at least 100 molecules for each of these pockets if not specified otherwise to strike a balance between sample size and compute time. The exact number of samples varies slightly as a consequence of the characteristics of the different methods. No statistical method was used to predetermine sample size.<br><br>Reference: Peng, Xingang, et al. "Pocket2mol: Efficient molecular sampling based on 3d protein pockets." International Conference on Machine Learning. PMLR, 2022.
Data exclusions	We excluded targets from the analysis for which one or several of the compared methods failed to generate samples.
Replication	The method contains elements that depend on pseudorandom numbers. We did not replicate results with different random seeds but ensure reproducibility by choosing a large sample size.
Randomization	The training/validation/test set split of the CrossDocked dataset was created in a previous work and is based on a 30% sequence similarity criterion. For BindingMOAD, we performed a random split that avoids overlap of the proteins' Enzyme Commission Number.<br>Both approaches aim to avoid fitting the overparameterized neural network models to data with high similarity to the final evaluation targets.
Blinding	The data splitting was performed in an automated way according to the procedure stated above.

Reporting for specific materials, systems and methods

We require information from authors about some types of materials, experimental systems and methods used in many studies. Here, indicate whether each material, system or method listed is relevant to your study. If you are not sure if a list item applies to your research, read the appropriate section before selecting a response.

Materials & experimental systems

n/a	Involved in the study
☒	☐ Antibodies
☒	☐ Eukaryotic cell lines
☒	☐ Palaeontology and archaeology
☒	☐ Animals and other organisms
☒	☐ Clinical data
☒	☐ Dual use research of concern

Methods

n/a	Involved in the study
☒	☐ ChIP-seq
☒	☐ Flow cytometry
☒	☐ MRI-based neuroimaging
